# Utilising a 1,8-naphthalimide probe for the ratiometric fluorescent visualisation of caspase-3

**DOI:** 10.3389/fchem.2024.1418378

**Published:** 2024-07-05

**Authors:** Conor Wynne, Robert B. P. Elmes

**Affiliations:** ^1^ Department of Chemistry, Maynooth University, National University of Ireland, Maynooth, Ireland; ^2^ Synthesis and Solid-State Pharmaceutical Centre (SSPC), Bernal Institute, University of Limerick, Castletroy, Ireland; ^3^ Kathleen Lonsdale Institute for Human Health Research, Maynooth University, National University of Ireland, Maynooth, Ireland

**Keywords:** caspase-3, ratiometric probe, naphthalimide, fluorescent sensor, peptide conjugate

## Abstract

The development of selective and sensitive probes for monitoring caspase-3 activity–a critical enzyme involved in apoptosis–remains an area of significant interest in biomedical research. Herein, we report the synthesis and characterisation of a novel ratiometric fluorescent probe, Ac-DEVD-PABC-Naph, designed to detect caspase-3 activity. The probe utilises a 1,8-naphthalimide fluorophore covalently linked to a peptide sequence via a self-immolative *p*-aminobenzyl alcohol (PABA) linker. Upon enzymatic cleavage by caspase-3, the probe undergoes spontaneous degradation, releasing the free naphthalimide fluorophore, resulting in a ratiometric change in fluorescence emission. Spectroscopic studies revealed a time-dependent ratiometric fluorescent response, demonstrating the probe’s ability to visualise caspase-3 activity with high sensitivity. Enzyme kinetics such as *K*_*m*_ (Michaelis constant), *k*_*cat*_ (turnover number), and *LOD* (Limit of Detection) were obtained, suggesting that the probe possesses comparable kinetic data to other probes in literature, but with the added benefits of ratiometric detection. Selectivity studies also demonstrated the probe’s specificity for caspase-3 over other endogenous species and enzymes. Ac-DEVD-PABC-Naph may be a promising tool for the quantitative detection and fluorescent visualisation of caspase-3 activity in biological systems, with potential applications in apoptosis research and drug development.

## 1 Introduction

Apoptosis was first coined by Kerr et al. while trying to detail a different form of cell death (Kerr et al., 1972). Since then, it has become clear that this morphologically-distinct mode of programmed cell death occurs during normal development of multicellular organisms to maintain healthy cell populations (Mehić, 2012). The regulation of apoptosis plays a vital role in various diseases such as cancer (Hanahan and Weinberg, 2011), Acquired Immunodeficiency Syndrome (AIDS) (Thompson Craig, 1995), Autoimmune Lymphoproliferative Syndrome (ALPS) (Chun et al., 2002), and neurodegenerative diseases like Parkinson’s and Alzheimer’s (Lin and Beal, 2006; Valko et al., 2007). Therefore, the monitoring of apoptosis within multicellular organisms is paramount to improve our understanding of these apoptosis-related diseases (Fischer and Schulze-Osthoff, 2005).

Caspase(s) 1–14 are a family of cysteine proteases that are important mediators of the apoptotic pathway (Shaulov-Rotem et al., 2016). The name “Caspase” was originally derived from a cysteine-dependant aspartate-specific protease, as reported by Alnemri et al., (1996) where cleavage of its substrate is regulated by a Cysteine side-chain (-SH) present on the enzyme, with an inherent (and strict) selectivity for severance on the *C*-terminal aspartic acid residue (Alnemri et al., 1996). Caspase-3 is widely regarded as the most proficient of the family, with a profoundly low *K*_*M*_ (Michaelis constant) and high *k*_*cat*_ (turnover number) for its substrates that preferentially cleaves any peptide moiety or proteins containing the sequence–DEVD (Denault and Salvesen, 2008). Thus, caspase-3 represents a useful biomarker to help gain an insight into the analysis of apoptosis.

Several strategies have been employed to monitor caspase-3 activity in recent years (Poręba et al., 2013; Vickers et al., 2013; Nicholls and Hyman, 2014). In particular, activity-based fluorescence probes have seen success where the use of latent fluorophore-conjugates coupled to the *C*-terminus of a DEVD peptide substrate can release a fluorescent reporter upon caspase-3 mediated cleavage (Gurtu et al., 1997; Wang et al., 2005; Kim et al., 2017). For example, Shi and coworkers have recently reported a DEVD probe conjugated to a hydrophobic Tetraphenylethene (TPE) fluorophore, with Aggregation-induced Emission (AIE) characteristics (Shi et al., 2012). This probe was virtually non-fluorescent in aqueous media, but experiences a dramatic surge in fluorescence intensity in response to caspase-3. Another popular strategy relies on the use of a dye/quencher pair motif which–when activated by caspase-3 – results in a “switch-on” of the dye fluorescence (Vuojola et al., 2012; Wu et al., 2020). Shaulov-Rotem and group have used this approach in their recent report on a quenched fluorescent activity-based probe (qABP) (Shaulov-Rotem et al., 2016). A blackberry quencher was incorporated to negate any fluorescent activity, and only emitting a fluorescent signal after covalent modification via caspase-3. Both of these strategies rely on the changes in fluorescence emission wavelength after enzymatic cleavage for analysis of activity. However, the accurate evaluation of caspase-3 activity *in cellulo* remains problematic, with many of the reported fluorogenic probes being affected by cellular localisation and microenvironments.

Ratiometric probes may provide an optimal solution to this problem owing to their advantages in quantitative detection (Dunn et al., 1994). The intensity of fluorescent light is heavily influenced by the quantity of fluorophore in the optical path. The amount of fluorophore in the optical path is determined by the actual concentration of the fluorophore within the cell (which is determined by marker uptake or expression) and the diameter of the specimen. Consequently, it is challenging to directly deduce the concentration of a particular species under investigation solely by observing one single fluorescence intensity (Chen et al., 2013). For instance, one cannot definitively state, “An intensity (a.u) of 100 corresponds to 100 nM of free calcium in a cell.” To address this challenge and enable accurate measurements of absolute species concentration(s), ratiometric imaging techniques have been developed. Ratiometric methods share a common approach in which the intensity of emitted light is measured twice, and a ratio of these intensities is calculated. In such methods, the fluorophore is typically excited with light of one wavelength, and the emitted light is measured at two different wavelengths. Indeed, this class of probes possess two distinct emission peaks that carry a self-calibration effect, which may adequately reduce many of the interferences mentioned above (He et al., 2017).

In addition to the aforementioned benefits, the calculation of a ratio provides an additional advantage. During live-cell imaging using fluorophores, minor fluctuations in fluorescence intensity at the respective wavelengths are often encountered. However, in ratio-imaging, it is common to observe an increase in intensity at one wavelength paired with a decrease in intensity at the other wavelength (regardless of whether the probe is excited or detected with two wavelengths). When the ratio of both acquired images is subsequently calculated, the difference between the baseline and signal-amplitude is accentuated, compared to the mere intensity change of the fluorophore. This enhances the sensitivity of detecting changes in the signal (Madhu et al., 2023).

**FIGURE 1:**
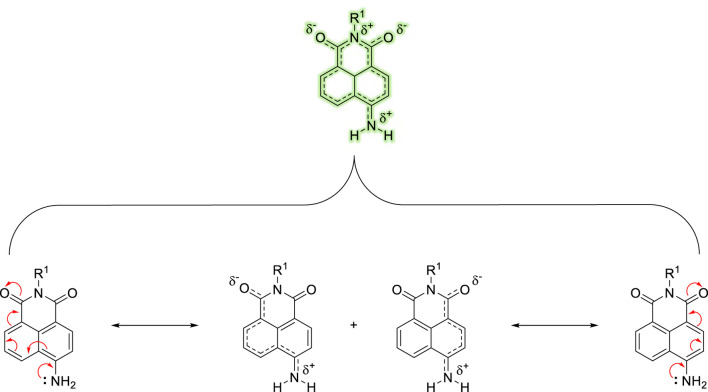
Illustration of the ICT within the 4-amino-1,8-naphthalimide fluorophore caused by an electronic “push−pull” system.

1,8-naphthalimides have been exploited to great effect in this regard where their tuneable photo-physics and synthetic versatility make them excellent candidates to elicit a ratiometric response (Duke et al., 2010; Banerjee et al., 2013). The naphthalimide offers two methods for detecting an analyte: through the conventional fluorescence “switch-on” response and, equally, by adjusting the Internal-Charge Transfer (ICT) excited state to produce a ratiometric fluorescence response. First documented by Middleton and colleagues in 1986 (Middleton et al., 1986), the 1,8-naphthalimide core exhibits a brightness similar to coumarins (Fu and Finney, 2018), along with remarkable resistance to photobleaching and a considerable Stokes shift. The photophysical properties of the naphthalimide fluorophore and its inherent characteristics are strongly impacted by the electronic nature of its substituents. For instance, the -NO_2_ functional group–when installed at the 4-position on the 1,8-naphthalimide core–triggers a broad absorption band with a λ_max_ ∼ 360 nm but exhibits minimal fluorescence. This is (primarily) due to the highly electronegative oxygen atoms that hinder the conjugation of the donor nitrogen to the rest of the fluorophore, thereby effectively obstructing its ICT. In contrast, the -NH_2_ derivative in the same position is “unrestricted” and elicits a “push-pull” ICT excited-state, leading to broad absorption and emission bands at approximately 450 and 550 nm, respectively (Figure 1). Furthermore, the amino-nitrogen enhances the ICT-nature of the fluorophore, causing the emission to red-shift towards longer wavelengths compared to less electron-donating substituents like esters and carbamates. Collectively, these properties impart numerous attributes suitable for biological applications, including assay design and the development of fluorogenic dyes for confocal microscopy. Notably, the capacity to fine-tune the ICT excited-state offers significant opportunities for exploitation.

**SCHEME 1:**
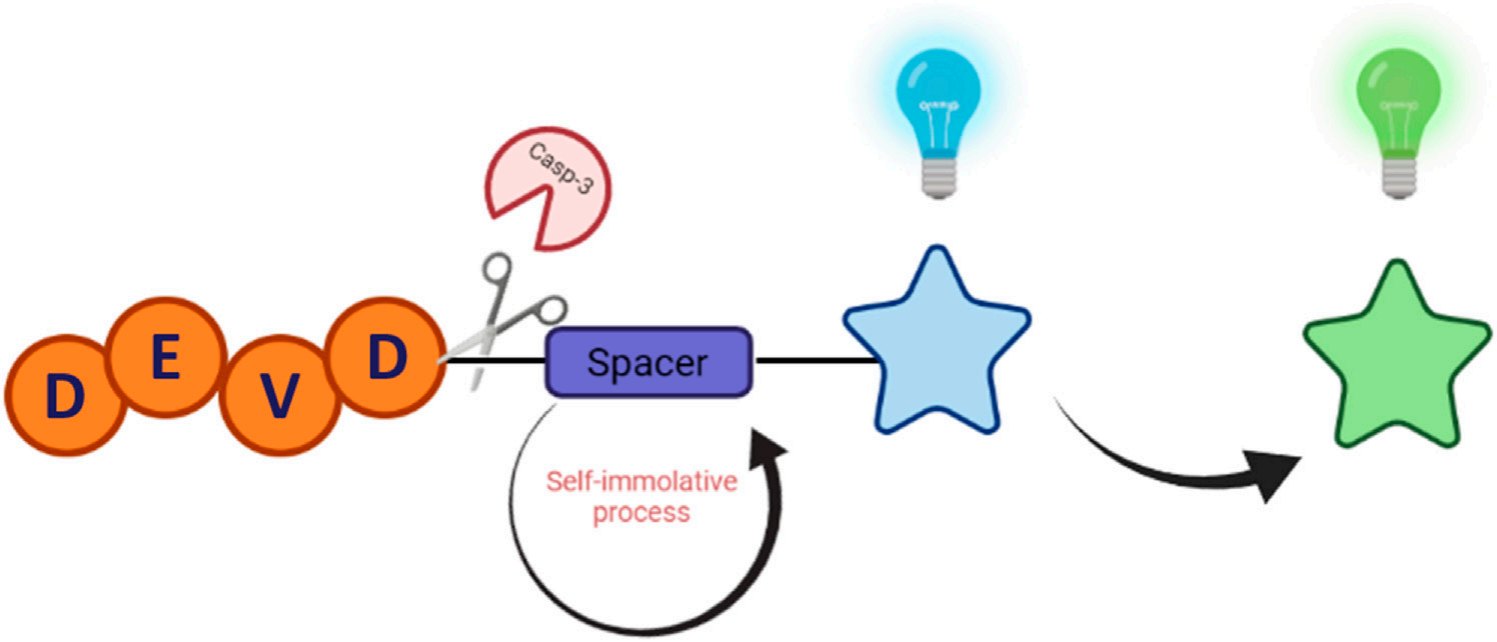
Cartoon schematic of ratiometric probe for the detection of Caspase-3.

Indeed, the ICT excited-state can be modulated effectively by functionalisation at the 4-position, and we (The Elmes group) have recently reported a 2-nitroimidazole-1,8-naphthalimide conjugate capable of detecting reductive stress in HeLa cells using a similar strategy. In this case, a clear blue to green ratiometric fluorescence response was observed upon reaction with anaerobic oxidoreductases and the impact of reductive stress could be easily monitored using confocal microscopy and flow cytometry (Ao et al., 2017). With these considerations in mind, we expected that a similar design strategy could be employed to effectively image caspase-3 activity and provide critical insight into the apoptotic pathway in living biological systems (Scheme 1).

**FIGURE 2:**
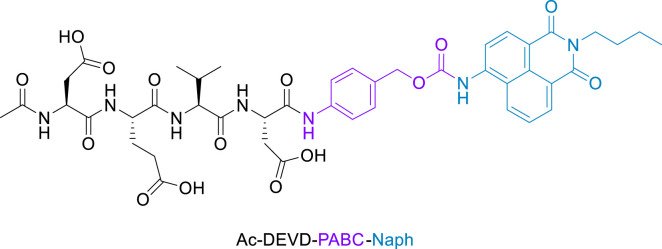
Structure of Fluorescent Caspase-3 probe (purple denotes the linker, and blue the fluorophore).

The objective of this work was to synthesize a peptide probe Ac-DEVD-PABC-Naph (Figure 2) that displayed a sensitive, selective and ratiometric fluorescence response for caspase-3. Ratiometric probes for the fluorescent visualisation of caspase-3 are rare, and are often complex molecules that require additional components–like cell-penetrating peptides–to achieve effectiveness (Jin et al., 2021).

**SCHEME 2:**
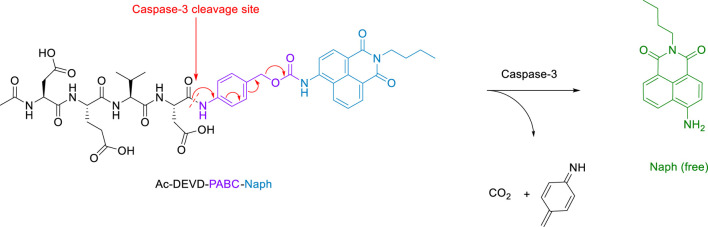
Schematic of Peptide Probe (Ac-DEVD-PABC-Naph) and its release mechanism for the ratiometric sensing of Caspase-3.

The essence of this strategy involves a cell-permeable 1,8-naphthalimide (Geraghty et al., 2021) (Naph) fluorophore covalently linked to an Ac-DEVD peptide sequence via a *p*-aminobenzyl alcohol (PABA) linker. We hope that this design will minimise any steric interactions between the cleavable bond and the bulky naphthalimide, while retaining the fast 1,6-elimination afforded by the PABA linker. Reaction with caspase-3 should give rise to a fragmentation of the parent molecule and release of the well-known amino-1,8-naphthalimide fluorophore (Scheme 2). It is expected that this elimination would lead to significant ICT modulation giving rise to the desired ratiometric response. Herein, we report the synthesis of Ac-DEVD-PABC-Naph and a detailed spectroscopic evaluation in response to caspase-3.

## 2 Experimental section

### 2.1 Synthesis of caspase-3 probe

The synthetic route to Ac-DEVD-PABC-Naph is a multi-step pathway involving several peptide-based intermediates before obtaining the deprotected final compound. The first step in the synthetic pathway was to assemble the protected (-O*t*Bu) DEVD sequence. This was completed using 2-Chlorotrityl Chloride resin, with sequential coupling via Fmoc/*t*Bu SPPS. After the first amino acid (Fmoc-Asp(O*t*Bu)-OH) was installed on the resin, it was then washed with a solution of DCM/MeOH/DIPEA to endcap any remaining reactive trityl groups. Estimation of the first residue attachment was achieved spectroscopically. The Fmoc group was then removed by a piperidine/DMF solution, with coupling of subsequent amino acids as follows; Fmoc-protected amino acids in the presence of PyBOP and *N*-methyl morpholine (NMM).

The completion of each coupling step was characterised via LC-MS. Upon coupling the last amino acid, the N-terminus of Aspartic Acid was capped with pyridine/acetic anhydride. Finally, the peptide was separated from the resin via a hexafluoroisopropanol (HFIP)/DCM solution, and the residual solvent was removed *in vacuo* before lyophilisation.

**SCHEME 3:**
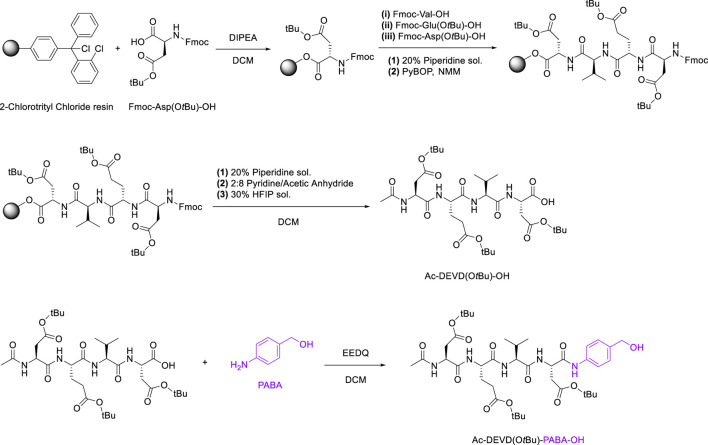
SPPS Synthetic Pathway for Ac-DEVD(O*t*Bu)-PABA-OH.

2-ethoxycarbonyl-2-ethoxy-1,2-dihydroquinoline (EEDQ) was added to dry DCM containing dissolved Ac-DEVD(O*t*Bu)-OH, and the reaction mixture was stirred for 20 min at room temperature. PABA was dissolved in dry DCM before being added to the previous solution via a syringe. The reaction was then allowed to stir for 16 h (Scheme 3), producing Ac-DEVD(O*t*Bu)-PABA-OH as a pale-yellow solid with a yield of 78%.

**SCHEME 4:**
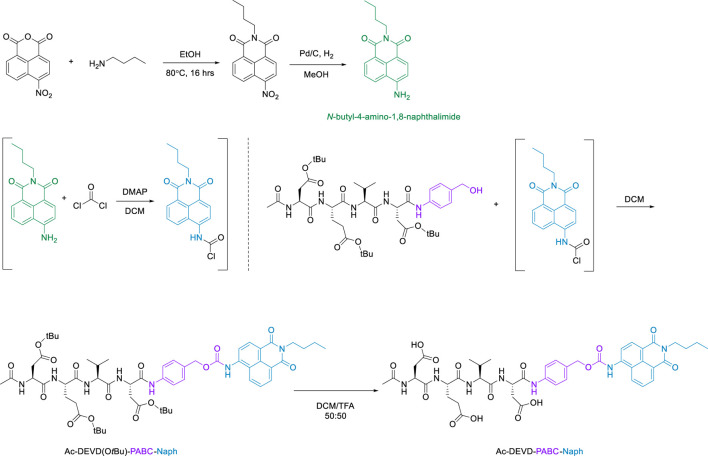
Functionalisation of Ac-DEVD(O*t*Bu)-PABA-OH with Naph fluorophore via carbamate linkage.

The final step in the synthetic-pathway was to functionalise the peptide-linker intermediate Ac-DEVD(O*t*Bu)-PABA-OH with the Naph fluorophore, through a carbamate linkage. This carbamate functional group breaks down to release CO_2_, and is a necessary component in the design strategy–as it allows the spontaneous release of the naphthalimide fluorophore after self-immolation of the PABC linker. In order to couple the fluorophore to the parent peptide-linker, the 4-amino-1,8-naphthalimide (Naph) was first converted to its corresponding carbamoyl chloride before reacting with the aliphatic alcohol on the PABA moiety (Scheme 4). This reaction proceeded until a precipitate had formed, which was washed and filtered to afford the protected product Ac-DEVD(O*t*Bu)-PABC-Naph with a yield of 66%.The protected peptide-conjugate was dissolved in TFA/DCM to facilitate deprotection of the -*t*Bu side-chains. This afforded the deprotected final product Ac-DEVD-PABC-Naph as a pale-yellow solid with a yield of 81%. LC-MS and HRMS experiments were used to characterise this final compound (see the ESI for full characterisation).

### 2.2 Caspase-3 enzymatic assay in solution

The enzymatic analysis was initially carried out with human recombinant caspase-3 *in vitro*. A stock solution of Ac-DEVD-PABC-Naph was prepared in dimethyl sulfoxide (DMSO). Ac-DEVD-PABC-Naph (10 µM) and caspase-3 were dissolved in assay buffer (50 mM HEPES, pH 7, 0.1% CHAPS, 10 mM DTT, 100 mM NaCl, 1 mM EDTA and 10% Sucrose) and 18 megohm water at 25°C in a quartz cuvette. The cuvette was placed into the cell holder, and fluorescence signals were measured with an excitation wavelength of 402 nm. The emission was collected from 410 to 750 nm. A blank solution without caspase-3 was also measured for comparison, under the same conditions.

## 3 Results and discussion

We have developed a versatile methodology for the synthesis of a caspase-3 selective ratiometric probe (Ac-DEVD-PABC-Naph). This strategy involved a naphthalimide fluorophore that was covalently bound to the peptide backbone via a PABC linker. A breakdown of our rationale is highlighted in Scheme 2. The 4-position on the naphthalimide core strongly governs its fluorescence profile. The amino-derivative (free Naph) in the same position is “unrestricted” and elicits a “push-pull” ICT excited-state, resulting in broad absorption and emission bands around 432 and 535 nm. With this in mind, Ac-DEVD-OH was designed to connect to Naph via PABC. An amide bond was formed between the *C*-terminal aspartic acid on Ac-DEVD-OH and the amino group within PABA. Upon enzymatic hydrolysis, PABC underwent spontaneous degradation via a 1,6-elimination, with the loss of CO_2_ and aza-quinone methide. The presence of H_2_O leads to rapid quenching of this high energy, highly electrophilic intermediate (Blencowe et al., 2011). This self-immolative process released the free Naph fluorophore, which produced a ratiometric change in fluorescence. In contrast to quantification of enzyme activity by fluorescent change at only one emission wavelength, the ratiometric change at two distinct wavelengths can quantitatively measure enzyme activity with increased accuracy.

### 3.1 Spectroscopic response to caspase-3

#### 3.1.1 UV-vis and fluorescence studies

**FIGURE 3:**
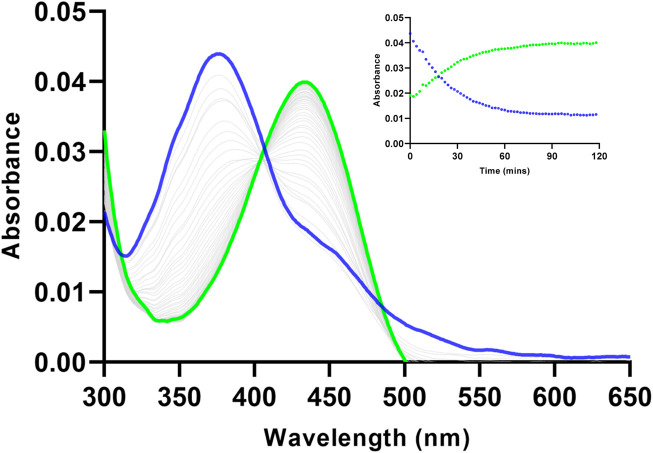
Changes observed in the absorbance spectrum of Ac-DEVD-PABC-Naph (10 µM) upon addition of Caspase-3 (200 ng/mL) in assay buffer (0.28% DMSO) pH 7.4, over 120 min. *Inset*: Absorbance intensities at 372 nm and 432 nm versus time.

The spectroscopic response of Ac-DEVD-PABC-Naph towards caspase-3 was investigated using a combination of UV-vis and fluorescence spectrophotometry. The changes in the absorbance spectrum (UV-vis) of Ac-DEVD-PABC-Naph (10 µM) were observed upon addition of caspase-3. The probe displayed a typical absorption peak at 372 nm before treatment with caspase-3 (200 ng/mL), which shifted to a peak maxima at 432 nm after 2 h. To gain an enhanced understanding of the changes observed in this absorption spectrum, the absorbance intensities were monitored at 5-min intervals for 2 h (Figure 3). Although the absorption intensities appeared quite low–which can sometimes be the case with naphthalimides (Martínez-Calvo et al., 2020; Geraghty et al., 2021) – there was still an obvious progression from the parent compound to the free Naph upon treatment with caspase-3 (200 ng/mL), with a clear isosbestic point at 402 nm.

**FIGURE 4:**
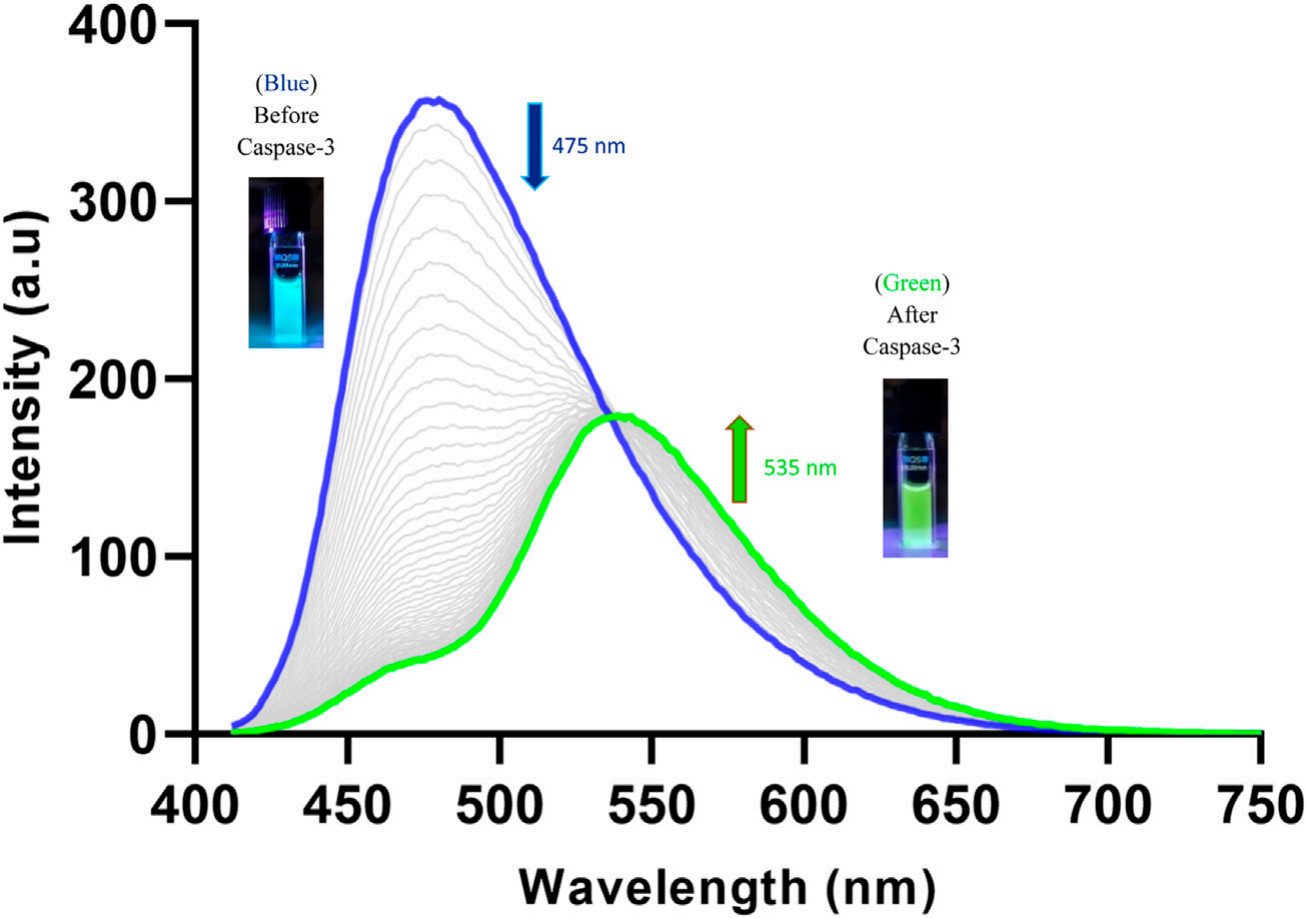
Changes observed in the fluorescence spectrum of Ac-DEVD-PABC-Naph (10 µM) upon addition of Caspase-3 (200 ng/mL) in assay buffer (0.28% DMSO) pH 7.4 over 120 min (λ_ex_ = 402 nm). *Inset:* Pictures of probe solutions with and without treatment of Caspase-3 under illumination via UV lamp.

Using this isosbestic point (402 nm) as the excitation wavelength, the probe displayed a maximum fluorescence emission at 475 nm (Figure 4). After treatment with caspase-3, the fluorescent peak at 475 nm diminished almost completely. A new emission peak corresponding to the free Naph appeared at 535 nm, suggesting that the probe was cleaved by caspase-3 to release the naphthalimide fluorophore. The ratiometric fluorescence signal (*I*_*535*_/*I*_*475*_) had increased over 2-fold after the probe was totally hydrolysed by caspase-3 (200 ng/mL), demonstrating the considerable potential of Ac-DEVD-PABC-Naph for the quantification of caspase-3.

**FIGURE 5:**
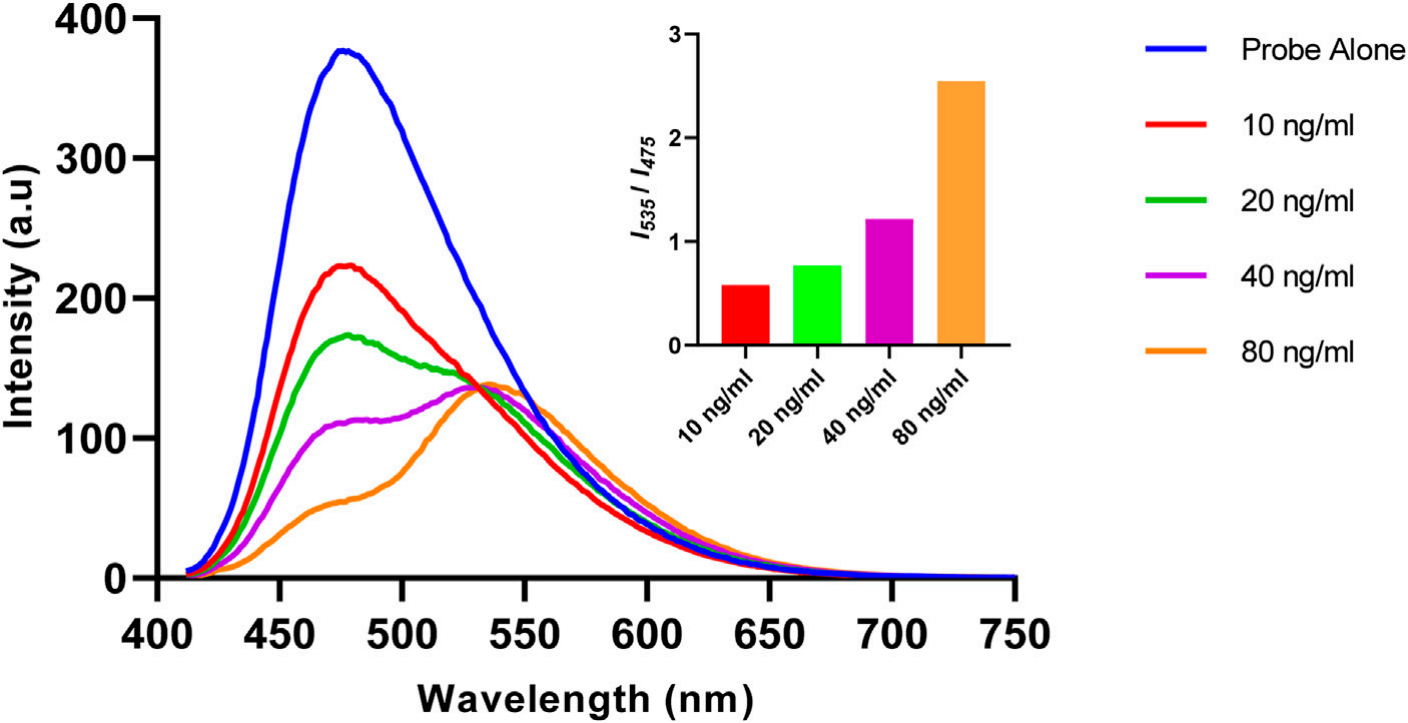
Fluorescence spectrum of Ac-DEVD-PABC-Naph (10 μM, 0.28% DMSO) in assay buffer, with various concentrations of Caspase-3 (λ_ex_ = 402 nm). *Inset:* Bar chart representing the fluorescent ratio response at λ_max_ 535 nm versus 475 nm.

The changes in the fluorescence profile of Ac-DEVD-PABC-Naph (10 µM) were then observed after treatment with known concentrations of caspase-3 (Figure 5). Increasing concentrations of caspase-3 (0–80 ng/mL) were used for this investigation, and a steady progression from the parent probe to the hydrolysed product was apparent. 10 ng of caspase-3 reduced the initial fluorescence output by roughly half. However, 10 and 20 ng/mL were unable to produce a ratiometric fluorescence signal (*I*_*535*_/*I*_*475*_) above 1.0. To achieve this, concentrations >40 ng/mL were required, with (unsurprisingly) the highest concentration of 80 ng/mL generating the best ratiometric response. This initial study gave us a brief insight into the enzyme kinetics associated with the reaction between caspase-3 and the probe.

The changes observed in the fluorescence profile of the probe proved our initial design strategy of the self-immolative pathway undertaken by the compound after treatment with caspase-3. The ICT excited-state of the free Naph appeared to be affected by the aqueous environment to a greater extent than the parent (un-hydrolysed) probe. The green fluorescence intensity maximum (535 nm) was roughly half that of the initial blue fluorescence (475 nm), even after 120 min. Although it is well-recorded in literature that the ICT-character of the substituent on the 4-position of the naphthalimide-core is very sensitive to its solvent environment (Gunnlaugsson et al., 2006; Duke et al., 2010), it is still worth noting that the carbamate functionality seems to provide some degree of *protection* in this regard. It’s postulated that when the 4-amino (-NH_2_) is functionalised with the carbamate linkage, its ICT to the rest of the naphthalimide fluorophore is modulated (Blue fluorescence in this case), but the intensity remains substantial–even in an aqueous environment.

This phenomenon was investigated by conducting Ionic Strength studies in aqueous media. As optical imaging is often done in buffered solutions, it is important to study the effect of ionic strength on the emission output of the probe. These experiments were performed with increasing additions of NaCl to an aqueous solution of Ac-DEVD-PABC-Naph (5 µM). There was only a small change in the fluorescence spectrum of the probe, with a slight decrease in fluorescence intensity observed when the concentration of NaCl was increased from 0 to 960 mM (Supplementary Figure S31). This study indicates that ionic strength does not affect the fluorescence output of Ac-DEVD-PABC-Naph.

**FIGURE 6:**
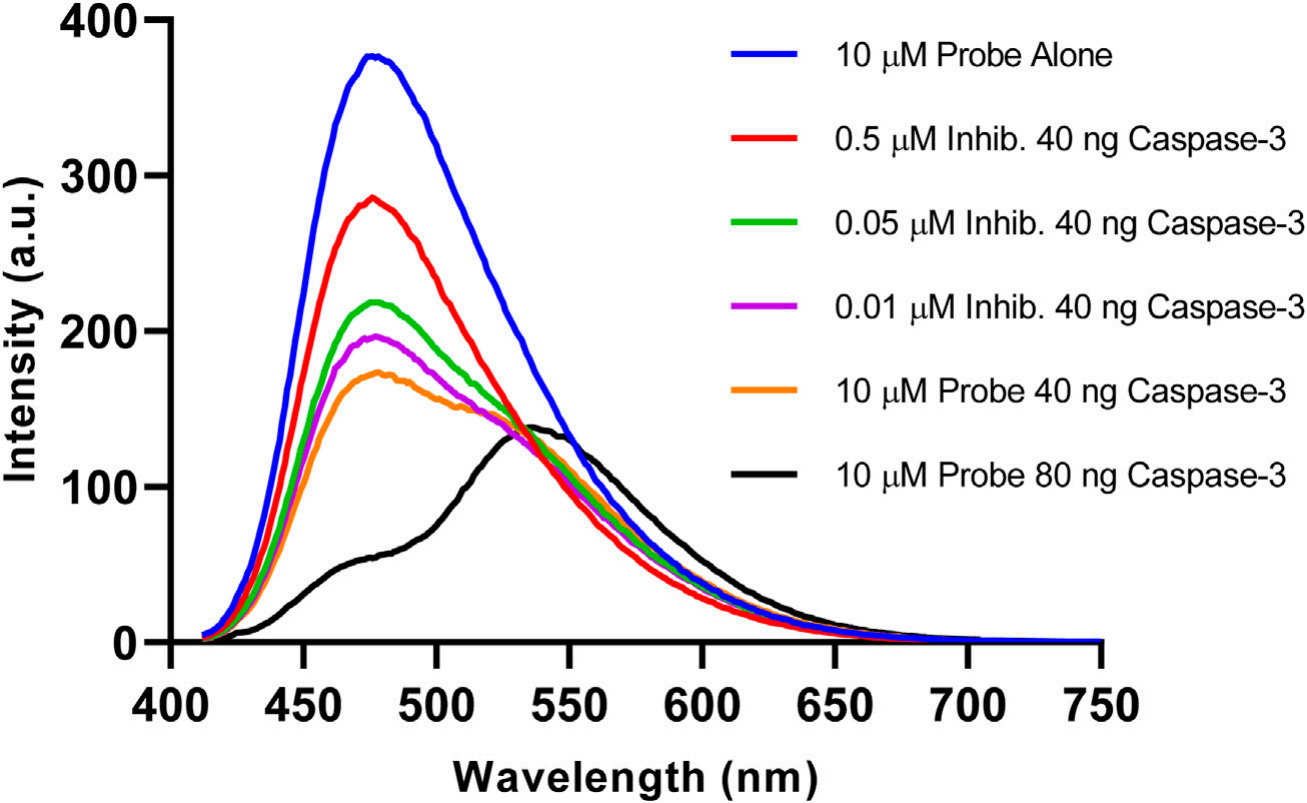
Fluorescence emission spectra (λ_ex_ = 402 nm) of reactions after incubation at 25°C for 1 h. (Blue) 10 µM probe, (Red) 10 µM probe + 0.5 µM Inhib. + 40 ng Caspase-3, (Green) 10 µM probe + 0.05 µM Inhib. + 40 ng Caspase-3, (Purple) 10 µM probe + 0.01 µM Inhib. + 40 ng caspase-3, (Orange) 10 µM probe + 40 ng Caspase-3, (Black) 10 µM probe + 80 ng Caspase-3, 3 h.

To investigate the role of caspase-3 in the fluorescence change of the probe, a potent reversible inhibitor for caspase-3 (Ac-DEVD-CHO) was added to the reaction mixture. As highlighted in Figure 6, the probe alone (Blue) showed no change in fluorescence over 1 h. However, when incubated with an increasing concentration of inhibitor, a partial loss of caspase-3 activity was observed with 0.05 µM (Purple) up to almost complete inactivity at 0.5 µM (Red). After the addition of 0.5 µM of inhibitor, the fluorescence signal at 475 nm only decreased slightly. This can be attributed to the almost complete loss in enzymatic activity of caspase-3. These results demonstrate the essential role of caspase-3 activity in changing the fluorescent profile of Ac-DEVD-PABC-Naph. Enzymatic cleavage of the probe by the active caspase-3 induces the ratiometric fluorescence response. Furthermore, the fluorescent ratio of the probe can be utilised for the visualisation of caspase-3 activity.

#### 3.1.2 Enzyme kinetics

**FIGURE 7:**
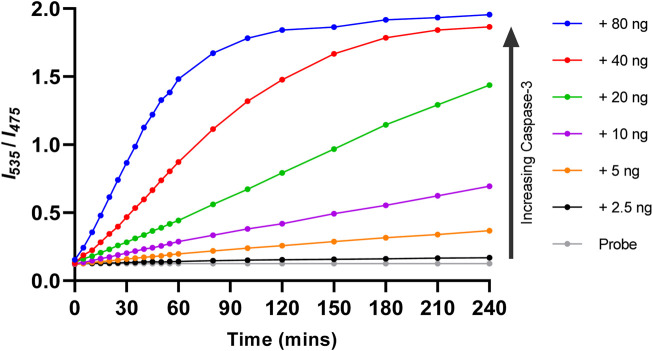
Response kinetics of Ac-DEVD-PABC-Naph (10 µM) towards different concentrations of capase-3 over 4 h.

Kinetic data for the reaction between Ac-DEVD-PABC-Naph (10 µM) and caspase-3 was generated by monitoring the changes in the ratiometric fluorescent signal (Figure 7). The fluorescence ratio (*I*_*535*_/*I*_*475*_) change, along with time, was documented with different known concentrations of caspase-3 (0–80 ng). In the absence of caspase-3 (Grey), the fluorescence ratio did not change over time, indicative of the probe’s stability in aqueous media. When caspase-3 was added, the probe was gradually hydrolysed, and the fluorescent ratio increased over a period of 4 h. Interestingly, it required upwards of 40 ng/mL (red and blue) concentrations of caspase-3 to achieve a complete plateau over the 4-h time period. In the first 30 min, the ratiometric fluorescence response of the probe to various concentrations of caspase-3 showed good linearity. Going forward, 30 min was used for the quantification of caspase-3.

**FIGURE 8:**
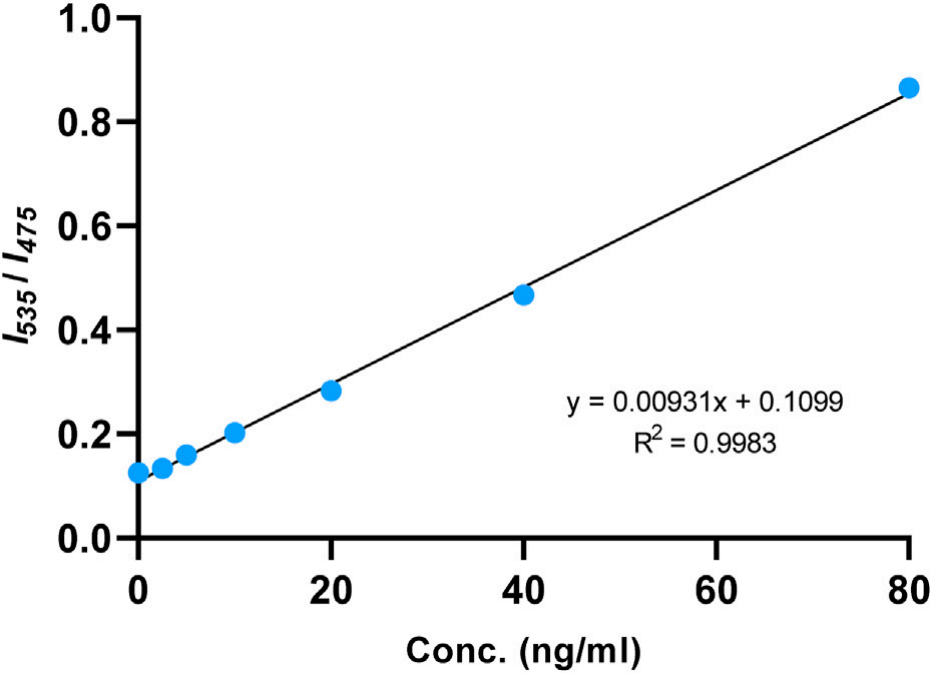
Linear fitting curve of fluorescence intensity ratio (*I*_*535*_ */I*_*475*_) to the concentration of caspase-3 (0–80 ng/mL). Reaction was incubated at 25°C for 30 min.

The fluorescence emission of Ac-DEVD-PABC-Naph with different concentrations of caspase-3 at 30 min was then extracted. The scatterplot of the data showed what appeared to be a linear relationship between the fluorescence ratio (*I*_*535*_/*I*_*475*_) and concentration of caspase-3. Fitting a straight-line to this data–linear regression–provided us with a mathematical model of this relationship that can be used to find the concentration (ng/mL) of caspase-3 in (potentially) any sample by obtaining its fluorescence ratio output. The fluorescence ratio (*I*_*535*_/*I*_*475*_) was indeed observed to increase linearly in the concentration range of 0–80 ng/mL of caspase-3 (Figure 8), with a coefficient of determination (*R* ^2^) equal to 0.9983 suggesting that the model provides an appropriate fit to the data.

The limit of detection (*LOD*) for caspase-3 using Ac-DEVD-PABC-Naph was calculated to be 4.96 ng/mL. This value was generated from a calibration curve (Supplementary Figure S32) detailing the fluorescence intensities at λ_max_ = 535 nm at known concentrations of caspase-3 (5–80 ng/mL). The *LOD* for this probe was higher than others reported in literature (Shi et al., 2012; Jin et al., 2021). Ratiometric probes for caspase-3 visualisation/quantification are rare, let alone naphthalimide-based ones, so direct comparison is difficult. The R^2^-value of the linear regression was slightly below the 0.990 threshold (Peris-Vicente et al., 2015), thus may warrant some degree of caution when interpreting data. Nevertheless, these results demonstrate the potential of the probe for the quantification of caspase-3 in aqueous media with high sensitivity.

The kinetic constants of caspase-3 toward Ac-DEVD-PABC-Naph were then evaluated to determine the effectiveness of this new ratiometric probe. In accordance with Michaelis-Menten kinetics, the initial rates of product formation under varying probe concentrations (1–10 µM) were monitored (Supplementary Figure S33). An expected trend was observed, with the lowest concentration (1 µM) requiring the least amount of time for complete hydrolysis–as evidenced by its quick time to plateau, compared to higher concentrations (10 µM) that took longer to reach complete hydrolysis of the substrate. This experiment was allowed to run for an extended period of 5 h to allow an adequate plateau region to form–which is necessary when computing kinetic data.

Once the data was fitted to the Michaelis-Menten equation *V*_*0*_ *= k*_*cat*_ *[E]*_*0*_ *[S]/(K*_*M*_ *+ [S])*, the kinetic constants were then calculated using a non-linear regression via *GraphPad™ Prism* ^*®*^ software (Lehninger et al., 2005). In this equation, the *K*_*M*_ of an enzyme-substrate (*ES*) complex is a numerical estimate of the affinity of a substrate for an enzyme, with the *k*_*cat*_ representing the number of catalytic cycles that each active-site can yield per unit time (s^−1^). The *K*_*M*_ and *k*_*cat*_ values of caspase-3 for Ac-DEVD-PABC-Naph were 46.4 µM and 0.51 s^−1^, respectively. These results suggests that the probe is slower to reach complete hydrolysis compared the literature standard Ac-DEVD-AFC (*K*_*M*_ = 12.7 µM, *k*_*cat*_ = 2.7 s^−1^) (Shi et al., 2012). This may be explained by the additional immolation process (of the PABC moiety) required by the probe once the *C*-terminal aspartic acid is hydrolysed by the enzyme. The larger *K*_*M*_ of the probe is not entirely unsurprising, as the bulky naphthalimide fluorophore may affect the substrate’s affinity for the enzyme.

#### 3.1.3 Selectivity studies

**FIGURE 9:**
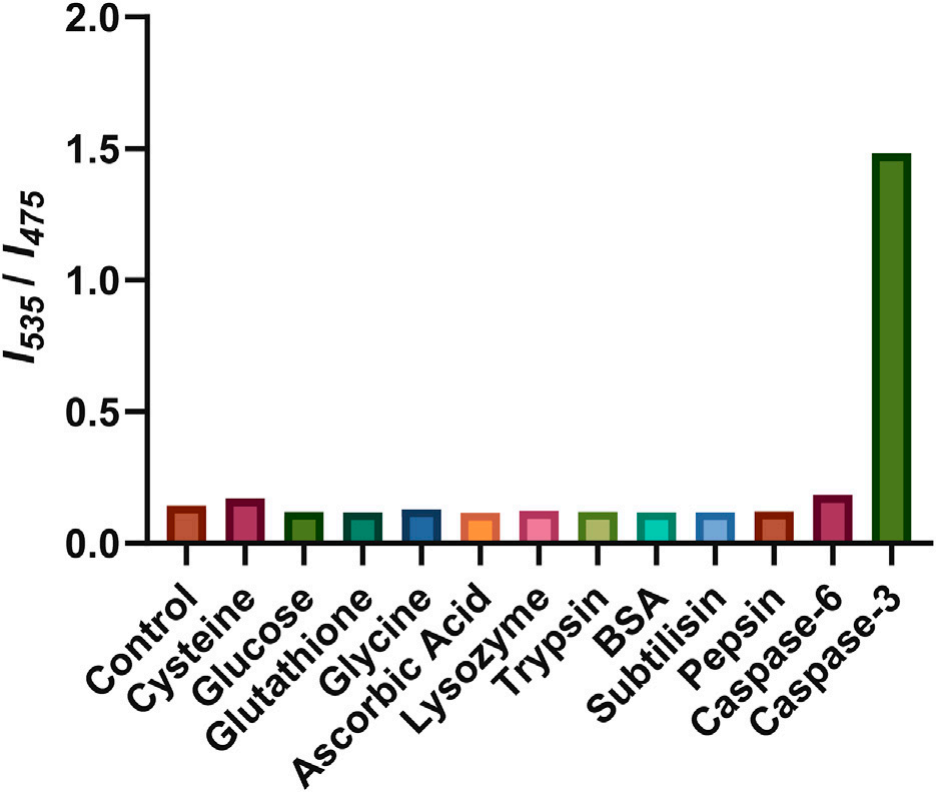
Fluorescence response of Ac-DEVD-PABC-Naph to various species. The concentrations of cysteine, glucose, glutathione, glycine, and ascorbic acid were all 10 mM. The concentrations of lysozyme, trypsin, BSA, subtilisin, and pepsin were all 1 μg/mL. The concentrations of caspase-3, and -6 are 80 ng/mL. Reaction time was 1 h at 25°C.

Finally, the selectivity of Ac-DEVD-PABC-Naph was explored to compare caspase-3 activity to other endogenous species, such as small biomolecules (cysteine, glucose, glutathione, glycine, and ascorbic acid) and proteins (lysozyme, trypsin, BSA, subtilisin, and pepsin). As depicted in Figure 9, none of the endogenous biomolecules could produce a fluorescence ratio change in the probe. Interestingly, none of the enzymes like lysozyme, trypsin, BSA, subtilisin, and pepsin could induce a fluorescence change either.

Only caspase-3 could induce a definite fluorescence ratio increase. Caspase-6, which is another member of the executioner caspases, was also investigated. Caspase-3 and -6 are believed to be responsible for the actual execution of cell death and often have short or absent pro-domains (Poręba et al., 2013). Caspase-6 has a preference for small hydrophobic side-chains like valine (**V**E*X*D) within its inherent substrate specificity. However, caspase-3 has a nearly absolute requirement for aspartic acid (**D**E*X*D), indicating its critical role in its enzymatic activity or substrate recognition. Indeed, the fluorescence ratio change observed for caspase-6 was far less than that produced by caspase-3. These results indicate the high selectivity of Ac-DEVD-PABC-Naph for caspase-3, therefore the ratiometric fluorescence signal could be employed as an indicator for caspase-3 activity.

## 4 Conclusion

Ac-DEVD-PABC-Naph displayed an absorbance band at 372 nm before the addition of caspase-3 and 432 nm after treatment with caspase-3. Likewise, a fluorescence emission maxima was observed at 475 nm for the unhydrolysed probe, and 535 nm after incubation with caspase-3. Both of these spectra clearly demonstrate the time-dependant ratiometric fluorescent response generated by the probe for the visualisation of caspase-3. Enzyme kinetics such as *K*_*m*_ (46.4 µM), *k*_*cat*_ (0.51 s^−1^), and *LOD* (4.96 ng/mL) were obtained using a non-linear regression via *GraphPad™ Prism* ^*®*^ software. These results indicate that the probe possesses comparable kinetic data to other probes in literature, but with the added benefits of ratiometric detection. The selectivity of the probe was demonstrated to be extremely adequate, with none of the endogenous biomolecules or enzymes (including caspase-6) generating a fluorescence ratio change.

We have demonstrated the ability of our probe to provide a ratiometric fluorescent response specifically to caspase-3, a key enzyme for the execution of apoptosis. Motivated by the selectivity and sensitivity of Ac-DEVD-PABC-Naph in response to caspase-3, we will conduct further experiments in regards to applying the probe for measuring caspase-3 activity in living cells.

## Data Availability

The original contributions presented in the study are included in the article/Supplementary Material, further inquiries can be directed to the corresponding author.
