# Supplementary figures and images for: Utilising a 1,8-naphthalimide probe for the ratiometric fluorescent visualisation of caspase-3
Source: Front Chem. 2024 Jul 5;12:1418378. doi: 10.3389/fchem.2024.1418378 (PMC11257929; doi:10.3389/fchem.2024.1418378)

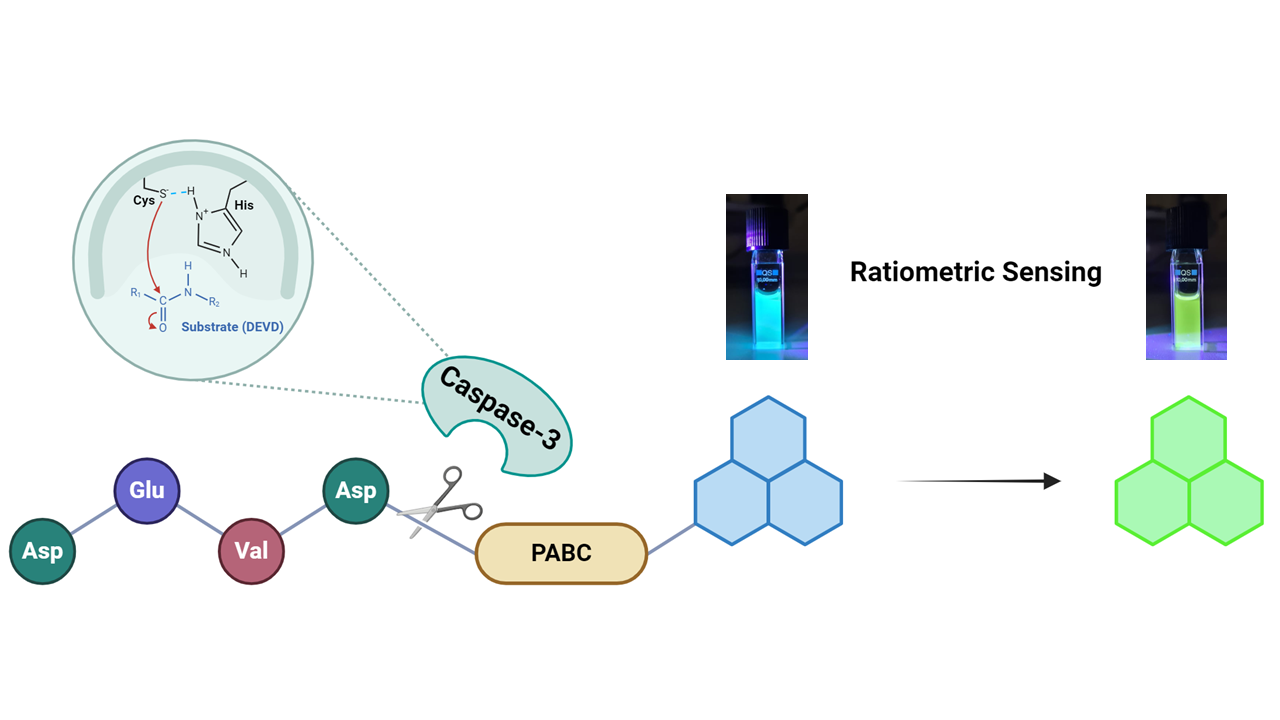

Supplement: Supplementary file 1 [file Image1.TIF]
