# Supplementary material for: Utilising a 1,8-naphthalimide probe for the ratiometric fluorescent visualisation of caspase-3
Source: Front Chem. 2024 Jul 5;12:1418378. doi: 10.3389/fchem.2024.1418378 (PMC11257929; doi:10.3389/fchem.2024.1418378)
Supplement: Supplementary file 2 [file DataSheet1.docx]

Supplementary Material

Utilising a 1,8-Naphthalimide Probe for the Ratiometric Fluorescent Visualisation of Caspase-3

Conor Wynne, ^1,2^ and Robert B. P. Elmes^*^ ^1,2,3^

^1.^ Department of Chemistry, Maynooth University, National University of Ireland, Maynooth, Co. Kildare, Ireland.

^2.^ Synthesis and Solid-State Pharmaceutical Centre (SSPC), University of Limerick, Bernal Institute, Castletroy, Co. Limerick, Ireland.

^3.^ Kathleen Lonsdale Institute for Human Health Research, Maynooth University, National University of Ireland, Co. Kildare, W23 F2H6 Maynooth, Ireland.

***Correspondence:**Robert Elmes,

Robert.Elmes@mu.ie

**Table of Contents**

[1 General Procedures and Instrumentation 2](#_Toc162292774)

[1.1 Loading of First Amino Acid Residue 2](#_Toc162292775)

[1.2 Sequential Amino Acid Coupling 3](#_Toc162292776)

[1.3 Cleavage of Amino Acid Sequence from Resin 3](#_Toc162292777)

[2 Experimental Details 3](#_Toc162292778)

[3 Characterisation Data 6](#_Toc162292779)

[4 Spectroscopic Data 23](#_Toc162292780)

[5 References 25](#_Toc162292781)

# General Procedures and Instrumentation

All reagents were of commercial quality. Solvents were dried and purified by standard methods – DCM was distilled over CaH_2_ and MeCN was dried over 3 Å molecular sieves. Anhydrous DMF was purchased from Sigma Aldrich. Analytical TLC was performed on aluminium sheets coated with a 0.2 mm layer of silica gel 60 F254. Silica gel 60 (230-400 mesh) was used for flash chromatography. Compounds were lyophilised on a Labconco Freezone 1 Dry system. LC-MS was performed on an Agilent Technologies 1200 series setup, utilising an Agilent Eclipse XDB-C18 (5µm, 4.6 x 150mm) column at 40°C. A flow rate of 0.2 ml min^-1^ and gradient of 0.1% of formic acid in CH_3_CN (solvent A) in 0.1% of formic acid in H_2_O (solvent B) was used as mobile phase. Electrospray in positive & negative mode was used for ionisation. NMR spectra were recorded using a Bruker Ascend 500 spectrometer, operated at 500 MHz for ^1^H NMR analysis and 126 MHz for ^13^C analysis, both at 293 K. The residual solvent peak was used as an internal standard for DMSO-*d_6_* and TMS for CDCl_3_. Chemical shifts (δ) were reported in ppm. NMR spectra were processed, and stack plots produced using MestReNova 6.0.2 software. The NMR spectra assignments were based on ^1^H, ^13^C, COSY, HSQC, and HMBC spectra. Multiplicity is given as s = singlet, bs = broad singlet, d = doublet, brd = broad doublet, dd = doublet of doublets, ddd= doublet of doublet of doublets, t = triplet, q = quartet, m = multiplet as appropriate, and *J* values are given in Hz. High resolution mass spectra (HRMS) were recorded – courtesy of Bath University – on an Agilent 6200 series TOF/6500 series Q-TOF instrument with an ESI source. UV-visible spectroscopy measurements were made at 25 ºC on a Lambda 365 Perkin Elmer UV-vis spectrophotometer. Fluorescence emission spectra were performed at 25 ºC on an Agilent Spectrofluorometer equipped with a 450 W xenon lamp for excitation. Starna and Hellma quartz cuvettes of 1 cm path length and several volumes were employed.

## Loading of First Amino Acid Residue

2-chlorotrityl chloride resin (*FluoroChem*) was weighed out into a 20 ml fritted-syringe. The resin was suspended in 10 ml of DCM and allowed to swell for ~1 hour. After this time, a solution of Fmoc amino-acid and DIPEA in DCM was added to the resin. The resin was agitated gently for 2 hours, then washed consecutively with *3x* DCM/MeOH/DIPEA (17:2:1), *3x* DCM, *3x* DMF, *3x* DCM, before being dried *in vacuo* (Athanassopoulos et al., 1995).

Dry Fmoc amino-acid resin (approx. 5 µmol with respect to Fmoc) was weighed into two separate 10 ml volumetric flasks. 2 ml of 10% Piperidine sol. (in DMF) was added and agitated gently for 30 minutes. The solution(s) were diluted to 10 ml with MeCN. 2 ml of each solution was transferred to two individual 25 ml volumetric flasks and diluted to the mark again with MeCN (solution 1 and solution 2, i.e. Duplicate). A reference solution was prepared in a similar manner as above, but without the addition of resin. Each cuvette was filled by taking 2.5 ml of each sample (Sol. 1, Sol. 2 & Ref.), before being placed into the spectrophotometer (Gude et al., 2002). The absorbance of each sample was recorded at 304 nm, and an estimate of first residue attachment was obtained from the following equation:

$$Fmoc Loading: mmol g^{-1}=\left( {Abs}_{sample\left( average \right)}-{Abs}_{Ref} \right) x \left( \frac{16.4}{{mg of resin}_{(average)}} \right)$$

## Sequential Amino Acid Coupling

The Fmoc amino-acid resin was swelled in DCM for ~30 minutes prior to Fmoc-deprotection. At the end of this time, the resin was treated with 3x 20% Piperidine sol. (DMF) for ~5 minutes, then subsequent washing with 3x DMF, 3x DCM and 3x DMF. A solution of Fmoc-AA, PyBOP and NMM in DMF was added to the resin and allowed to shake for 90 minutes. The new AA-resin was then washed with 3x DCM, 3x DMF and 3x DCM, before being dried in vacuo.

## Cleavage of Amino Acid Sequence from Resin

A very small spatula tip amount of dry Fmoc-AA resin was placed into a suitable vial, and then treated with a 30% HFIP/DCM solution (1 ml) for 90 minutes (with gentle agitation). After this time, the cleavage solution and resin were filtered through a glass pasteur pipette fitted with cotton wool. The HFIP/DCM was removed from the filtrate via evaporation with compressed air. The residue that remained was redissolved in MeCN prior to LC-MS analysis (Merck, 2024).

# Experimental Details

**Fmoc-VD(O*t*Bu)**

A solution of Fmoc-Val-OH (390 mg, 1.14 mmol, 3 eq.), PyBOP (1.19 g, 2.28 mmol) and NMM (376 µl, 3.42 mmol) in DMF (8 ml) was stirred in a small beaker for 15 mins. After this time, the Fmoc-AA solution was added to the resin and allowed to shake for 90 mins. The new peptide resin was then washed with *3x* DCM, *3x* DMF and *3x* DCM. **LC-MS** (0-100%) gradient of A in B, 55 min) *t*_R_ = 38 min.

**Fmoc-DEVD(O*t*Bu)**

Fmoc deprotection and test cleavage as before. A solution of Fmoc-Asp(O*t*Bu)-OH (470 mg, 1.14 mmol, 3 eq.), PyBOP (1.19 g, 2.28 mmol) and NMM (376 µl, 3.42 mmol) in DMF (8 ml) was stirred in a small beaker for 15 mins. After this time, the Fmoc-AA solution was added to the resin and allowed to shake for 90 mins. The new peptide resin was then washed with *3x* DCM, *3x* DMF and *3x* DCM. **LC-MS** (0-100%) gradient of A in B, 55 min) *t*_R_ = 42 min.

**Ac-DEVD(O*t*Bu)-OH**

(2*S*,5*S*,8*S*,11*S*)-11-acetamido-2-(2-(*tert*-butoxy)-2-oxoethyl)-8-(3-(*tert*-butoxy)-3-oxopropyl)-5-isopropyl-15,15-dimethyl-4,7,10,13-tetraoxo-14-oxa-3,6,9-triazahexadecanoic acid

Fmoc deprotection as before. The peptide resin was treated with a solution of Pyridine/Acetic Anhydride (2:8, 8 ml) and allowed to shake for 30 mins. The new resin was washed with *3x* DCM, *3x* DMF and *3x* DCM. LC-MS indicated complete consumption of starting material, with the complete loss of UV activity (loss of chromophore). Ninhydrin Test was used to confirm that the *N*-Terminus was completely acetylated (no primary amine present). The product was then removed from the resin via 30% HFIP/DCM (as before), to afford the *N*-Terminal *protected linear peptide* (**Ac-DEVD(O*t*Bu)-OH**) as an off-white solid – without further purification (227 mg, 87 %). **^1^H NMR** (500 MHz, CDCl_3_) δ 8.20 (d, *J* = 8.2 Hz, 1H), 8.09 (d, *J* = 7.2 Hz, 1H), 7.90 (d, *J* = 8.1 Hz, 1H), 7.78 (d, *J* = 9.0 Hz, 1H), 4.62 – 4.53 (m, 1H), 4.52 – 4.42 (m, 1H), 4.35 – 4.25 (m, 1H), 4.20 – 4.10 (m, 1H), 2.65 – 2.58 (m, 1H), 2.40 (d, *J* = 7.4 Hz, 2H), 2.23 – 2.10 (m, 2H), 1.99 – 1.91 (m, 1H), 1.91 – 1.84 (m, 1H), 1.83 (s, 3H), 1.76 – 1.63 (m, 1H), 1.37 (s, 27H), 0.82 (d, *J* = 6.8 Hz, 6H).

**Ac-DEVD(O*t*Bu)-PABA-OH**

*tert*-butyl-(4*S*,7*S*,10*S*,13*S*)-4-(2-(*tert*-butoxy)-2-oxoethyl)-7-(3-(*tert*-butoxy)-3-oxopropyl)-13-((4-(hydroxymethyl)phenyl)carbamoyl)-10-isopropyl-2,5,8,11-tetraoxo-3,6,9,12-tetraazapentadecan-15-oate

The *protected* *linear peptide* (150 mg, 0.22 mmol) was dissolved in a solution of DCM (10 ml) containing EEDQ (163 mg, 0.66 mmol). A few drops of DMSO were added to aid dissolution, along with 10 mins of sonication. This mixture was then allowed to stir for 20 mins under N_2_-atmosphere at room temp. After this time, PABA (54 mg, 0.44 mmol) was added, and the new solution was stirred for 16 hrs. Reaction progress was monitored via LC-MS, with the appearance of a UV-peak resembling the product. The organic layer was extracted from subsequent washes with 5% Acetic Acid (aq.) & H_2_O. The crude mixture was then re-dissolved in a minimum amount of MeCN before being purified via reverse-phase flash chromatography using a 0-50% gradient of MeCN in H_2_O as eluent, to afford **Ac-DEVD(O*t*Bu)-PABA-OH** as a pale-yellow solid (136 mg, 78 %). **LC-MS** (0-100%) gradient of A in B, 55 min) *t*_R_ = 28 min. **^1^H NMR** (500 MHz, DMSO) δ 9.85 (s, 1H), 8.34 (d, *J* = 7.0 Hz, 1H), 8.20 (d, *J* = 8.1 Hz, 1H), 7.95 (d, *J* = 7.5 Hz, 1H), 7.82 (d, *J* = 8.5 Hz, 1H), 7.54 (d, *J* = 8.6 Hz, 2H), 7.23 (d, *J* = 8.6 Hz, 2H), 5.11 (t, *J* = 6.9 Hz, 1H), 4.73 – 4.67 (m, 1H), 4.60 – 4.55 (m, 1H), 4.42 (s, 2H), 4.33 – 4.26 (m, 1H), 4.15 – 4.10 (m, 1H), 2.74 (dd, *J* = 15.8, 6.4 Hz, 1H), 2.62 (dd, *J* = 16.0, 5.6 Hz, 1H), 2.54 (dd, *J* = 15.8, 8.1 Hz, 1H), 2.41 (dd, *J* = 16.0, 8.6 Hz, 2H), 2.24 – 2.14 (m, 2H), 1.97 – 1.92 (m, 1H), 1.89 – 1.85 (m, 1H), 1.83 (s, 3H), 1.76 – 1.67 (m, 1H), 1.36 (s, 27H), 0.82 (d, *J* = 6.8 Hz, 6H). **^13^C NMR** (126 MHz, DMSO) δ 171.8, 171.0, 170.7, 170.6, 169.4, 169.3, 169.0, 168.7, 150.5, 137.6, 137.4, 136.1, 129.5, 128.9, 128.1, 127.9, 127.0, 126.9, 126.6, 121.5, 119.1, 80.3, 80.15, 79.6, 62.6, 57.8, 51.9, 50.5, 49.4, 39.5, 37.3, 31.2, 30.5, 27.8, 27.7, 27.7, 27.3, 22.5, 19.1, 18.1.

**Nitro Naphthalimide**

2-butyl-6-nitro-1*H*-benzo[*de*]isoquinoline-1,3(2*H*)-dione

*N*-butyl amine (794 µl, 8.04 mmol) was added to a solution of 4-nitro naphthalic anhydride (1.63 g, 6.70 mmol) in EtOH (15 ml). The reaction was heated to reflux and allowed to stir for 16 hrs. TLC indicated complete consumption of starting material. The EtOH was removed under reduced pressure and the crude mixture was re-dissolved in a minimum amount of DCM, before being purified via flash chromatography using a 0-10% gradient of MeOH in DCM as eluent, to afford the **nitro naphthalimide** as a light-brown solid (1.68 g, 84 %). **LC-MS** (0-100%) gradient of A in B, 55 min) *t*_R_ = 32 min. **^1^H NMR** (500 MHz, DMSO) δ 8.69 (d, *J* = 8.7 Hz, 1H), 8.63 – 8.61 (m, 1H), 8.59 (d, *J* = 8.0 Hz, 1H), 8.54 (d, *J* = 8.0 Hz, 1H), 8.08 (dd, *J* = 8.6, 7.4 Hz, 1H), 4.07 – 4.01 (m, 2H), 1.67 – 1.59 (m, 2H), 1.41 – 1.32 (m, 2H), 0.93 (t, *J* = 7.4 Hz, 3H). **^13^C NMR** (126 MHz, DMSO) δ 162.9, 162.1, 149.1, 131.7, 130.1, 129.6, 128.7, 128.4, 126.7, 124.2, 122.8, 122.7, 39.5, 29.5, 19.8, 13.7.

**Amino Naphthalimide (Naph)**

6-amino-2-butyl-1*H*-benzo[*de*]isoquinoline-1,3(2*H*)-dione

Nitro naphthalimide (1.68g, 5.63 mmol) was added to a suspension of Pd/C (120 mg, 1.13 mmol) in MeOH (25 ml). The mixture was bubbled through with H_2_ gas for 10 mins before leaving to stir under a H_2_-atmosphere for 16 hours. TLC indicated complete consumption of starting material. After this time, the reaction mixture was filtered through a pad of *Celite^®^* (SiO_2_) and washed with excess MeOH to remove the Pd/C. The MeOH filtrate was then removed under reduced pressure to yield the **amino naphthalimide** – without further purification – as a deep orange solid (1.43 g, 95 %). **LC-MS** (0-100%) gradient of A in B, 55 min) *t*_R_ = 25 min. **^1^H NMR** (500 MHz, DMSO) δ 8.60 (d, *J* = 8.4 Hz, 1H), 8.41 (d, *J* = 7.2 Hz, 1H), 8.18 (d, *J* = 8.4 Hz, 1H), 7.66 – 7.61 (m, 1H), 7.42 (s, 2H), 6.83 (d, *J* = 8.4 Hz, 1H), 4.03 – 3.97 (m, 2H), 1.61 – 1.53 (m, 2H), 1.37 – 1.27 (m, 2H), 0.91 (t, *J* = 7.4 Hz, 3H). **^13^C NMR** (126 MHz, DMSO) δ 163.8, 162.9, 152.7, 133.9, 131.0, 129.7, 129.3, 124.0, 121.8, 119.4, 108.1, 107.6, 39.5, 29.8, 19.8, 13.7.

**Ac-DEVD(O*t*Bu)-PABC-Naph**

*tert*-butyl-(4*S*,7*S*,10*S*,13*S*)-4-(2-(*tert*-butoxy)-2-oxoethyl)-7-(3-(*tert*-butoxy)-3-oxopropyl)-13-((4-((((2-butyl-1,3-dioxo-2,3-dihydro-1*H*-benzo[*de*]isoquinolin-6-yl)carbamoyl)oxy)methyl)phenyl)carbamoyl)-10-isopropyl-2,5,8,11-tetraoxo-3,6,9,12-tetraazapentadecan-15-oate

Amino naphthalimide (9.6 mg, 0.036 mmol) was added to a solution of DMAP (11 mg, 0.09 mmol) in dry DCM (30 ml), and the solution was stirred at -10 °C in a salt/ice bath for 15 mins under a N_2_-atmosphere. A 15% Phosgene in Toluene sol. (12.9 µl, 0.18 mmol) was added dropwise – using a glass syringe and needle – over the course of 10 mins. Once all the phosgene solution had been added, the reaction mixture was allowed to stir for 30 mins in the ice-bath, then another 4 hrs at room temperature while still under N_2_-atmosphere. After this time, a complete change in fluorescence – from green to blue – was observed (UV torch). The DCM was then removed via compressed N_2_ gas, until complete dryness. A solution of Ac-DEVD(O*t*Bu)-PABA-OH (25 mg, 0.03 mmol) in DCM (15 ml) was added dropwise via syringe over the (now dry) reaction mixture, and was allowed to stir at room temp. under N_2_-atmosphere for 16 hrs. The precipitate that formed was filtered and washed with excess DCM to yield the protected product **Ac-DEVD(O*t*Bu)-PABC-Naph** – without further purification – as a light yellow solid (21.8 mg, 66 %). **LC-MS** (0-100%) gradient of A in B, 55 min) *t*_R_ = 44 min. **^1^H NMR** (500 MHz, DMSO) δ 10.37 (s, 1H), 10.00 (s, 1H), 8.70 (d, *J* = 8.7 Hz, 1H), 8.49 (dd, *J* = 13.7, 7.3 Hz, 2H), 8.37 (d, *J* = 7.7 Hz, 1H), 8.20 (d, *J* = 8.2 Hz, 2H), 7.96 (d, *J* = 7.9 Hz, 1H), 7.85 – 7.80 (m, 2H), 7.65 (d, *J* = 8.6 Hz, 2H), 7.43 (d, *J* = 8.6 Hz, 2H), 5.21 (s, 2H), 4.75 – 4.68 (m, 1H), 4.60 – 4.54 (m, 1H), 4.33 – 4.27 (m, 1H), 4.16 – 4.11 (m, 1H), 4.03 (t, *J* = 7.4 Hz, 2H), 2.75 (dd, *J* = 15.8, 6.3 Hz, 1H), 2.61 (dd, *J* = 16.1, 5.6 Hz, 1H), 2.58 – 2.53 (m, 1H), 2.41 (dd, *J* = 15.9, 8.6 Hz, 1H), 2.26 – 2.11 (m, 2H), 1.98 – 1.90 (m, 1H), 1.90 – 1.85 (m, 1H), 1.83 (s, 3H), 1.76 – 1.67 (m, 1H), 1.64 – 1.57 (m, 2H), 1.36 (s, 27H), 1.23 (s, 2H), 0.92 (t, *J* = 7.4 Hz, 3H), 0.82 (d, *J* = 6.9 Hz, 6H). **^13^C NMR** (126 MHz, DMSO) δ 171.8, 171.0, 170.7, 170.6, 169.3, 168.99, 168.9, 163.5, 163.0, 154.0, 140.7, 138.8, 131.7, 131.1, 130.9, 129.3, 129.1, 128.4, 126.4, 123.9, 122.3, 119.2, 118.2, 117.1, 80.4, 80.1, 79.6, 66.4, 57.8, 51.9, 50.5, 49.4, 39.5, 37.3, 31.2, 30.5, 29.7, 27.8, 27.7, 27.7, 27.3, 22.5, 19.8, 19.0, 18.1, 13.7. **HRMS** (ESI) *m/z:* Calc. for C_56_H_75_N_7_O_15_ ([M+H]^+^): 1086.5321, Found: 1086.5361

**Ac-DEVD-PABC-Naph deprotected**

(4*S*,7*S*,10*S*,13*S*)-13-((4-((((2-butyl-1,3-dioxo-2,3-dihydro-1*H*-benzo[*de*]isoquinolin-6-yl)carbamoyl)oxy)methyl)phenyl)carbamoyl)-7-(2-carboxyethyl)-4-(carboxymethyl)-10-isopropyl-2,5,8,11-tetraoxo-3,6,9,12-tetraazapentadecan-15-oic acid

*Protected peptide-conjugate* (21 mg, 0.02 mmol) was dissolved in 50:50 TFA/DCM (4 ml) and allowed to stir at r.t.p for 1 hour. LC-MS indicated the complete consumption of starting material. Thus, the TFA/DCM was evaporated using compressed air. The resulting oil was re-dissolved in a minimum amount of MeCN before being purified via reverse-phase flash chromatography using a 0-5% gradient of MeCN in H_2_O as eluent, to afford **Ac-DEVD-PABC-Naph** as a pale yellow solid (15 mg, 81 %). **LC-MS** (0-100%) gradient of A in B, 55 min) *t*_R_ = 32 min. **HRMS** (ESI) *m/z:* Calc. for C_44_H_51_N_7_O_15_ ([M+H]^+^): 918.3443, Found: 918.3502

# Characterisation Data


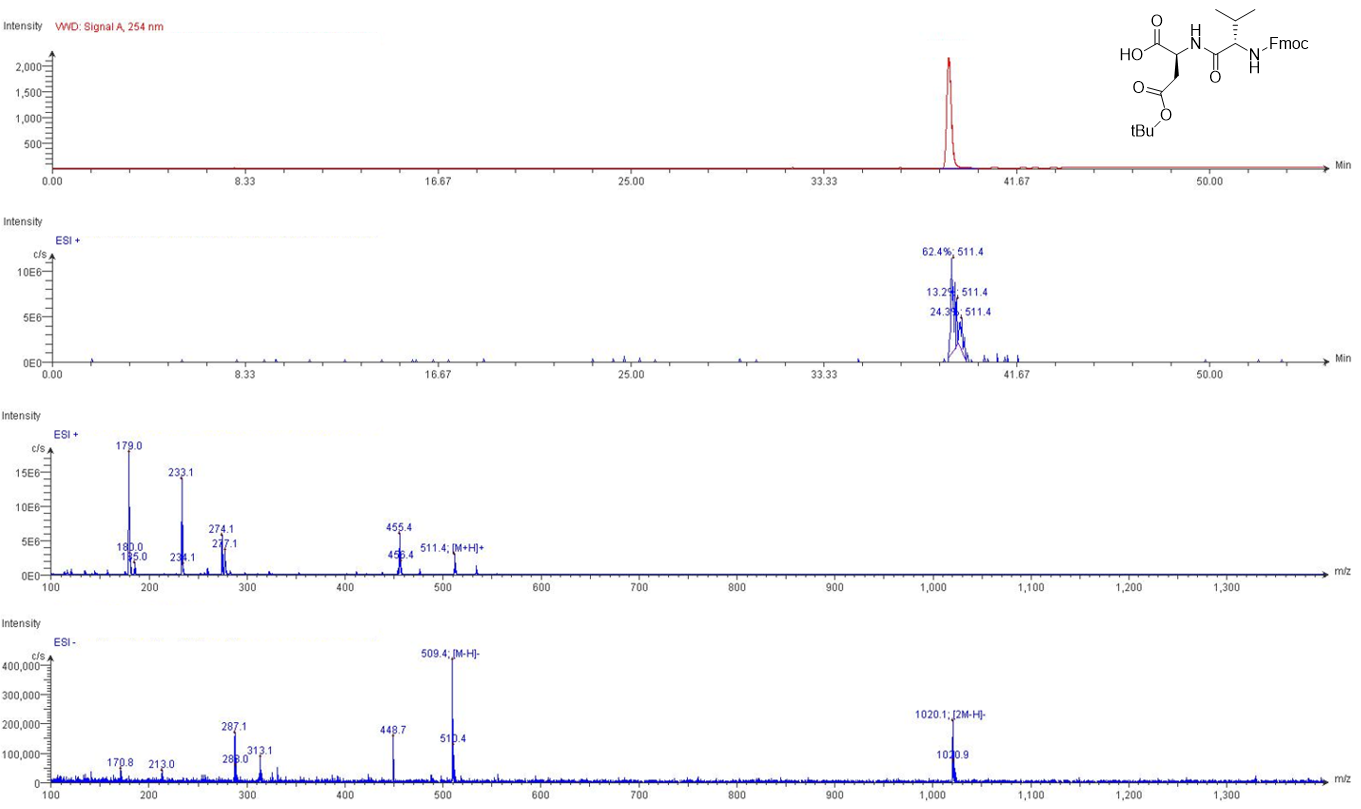


**Figure S1**: LC-MS data for **Fmoc-VD(O*t*Bu)-OH**.


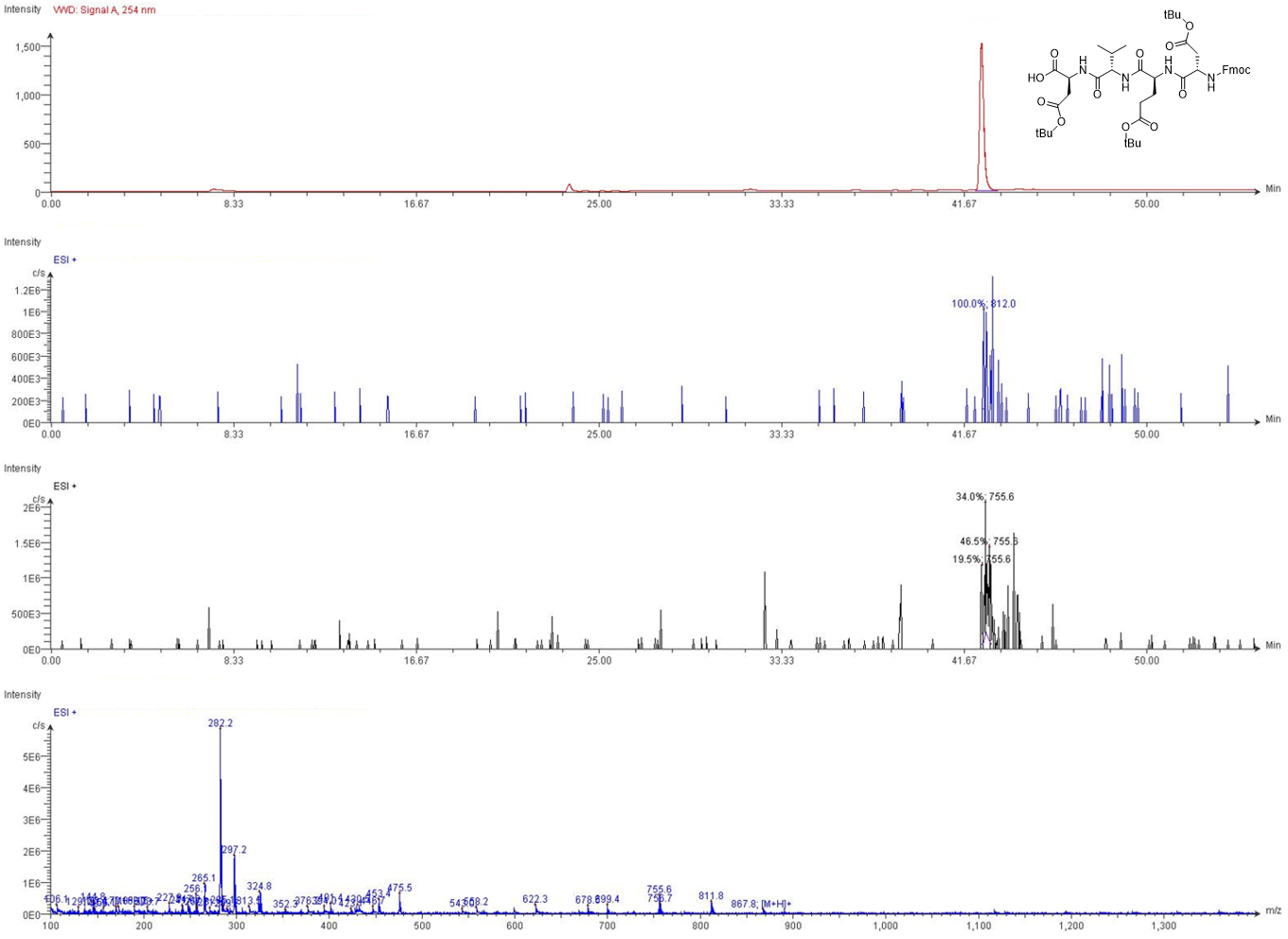


**Figure S2:** LC-MS data for **Fmoc-DEVD(O*t*Bu)-OH**.


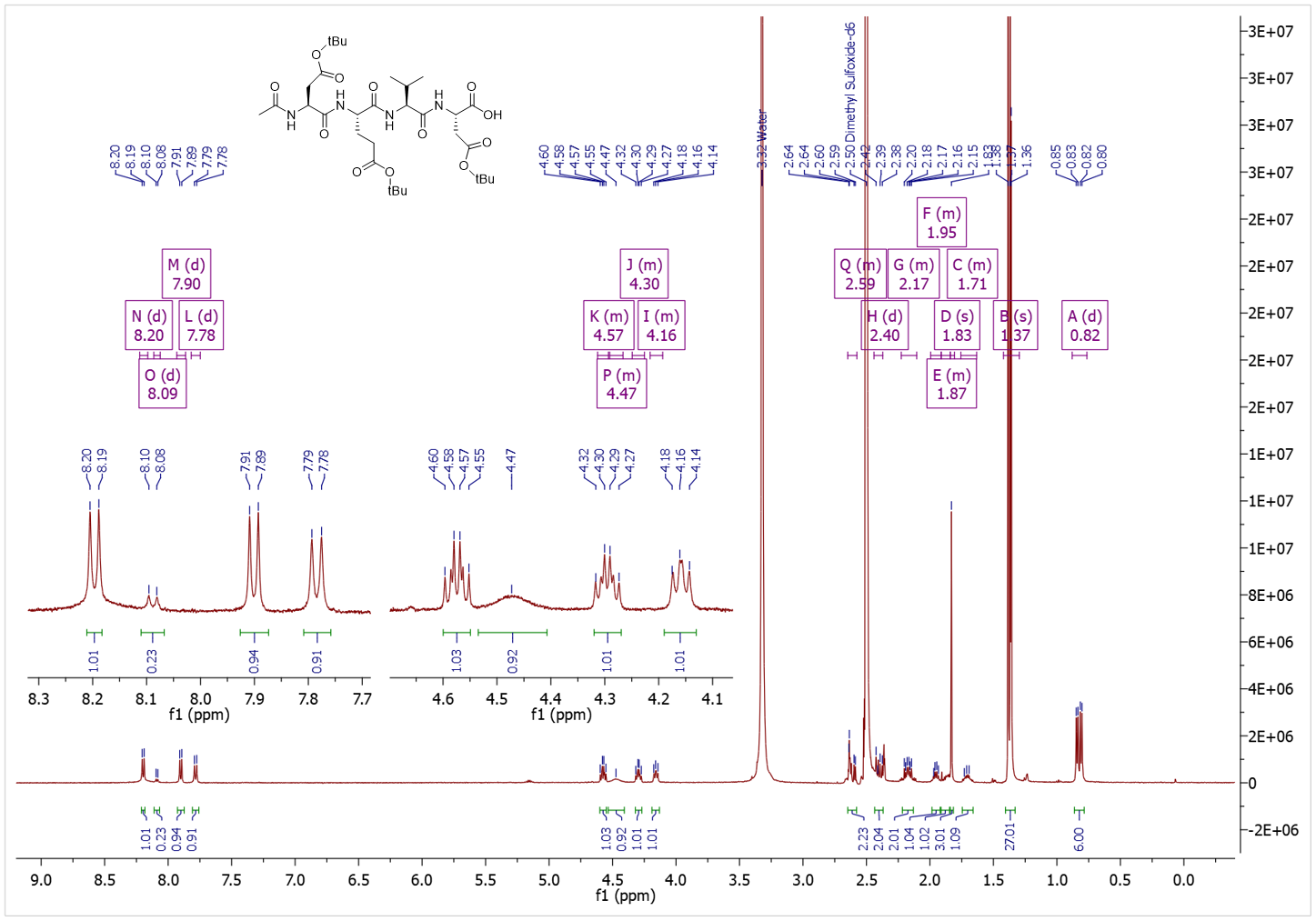


**Figure S3:** ^1^H NMR spectrum of **Ac-DEVD(O*t*Bu)-OH** in DMSO-*d*_6_.


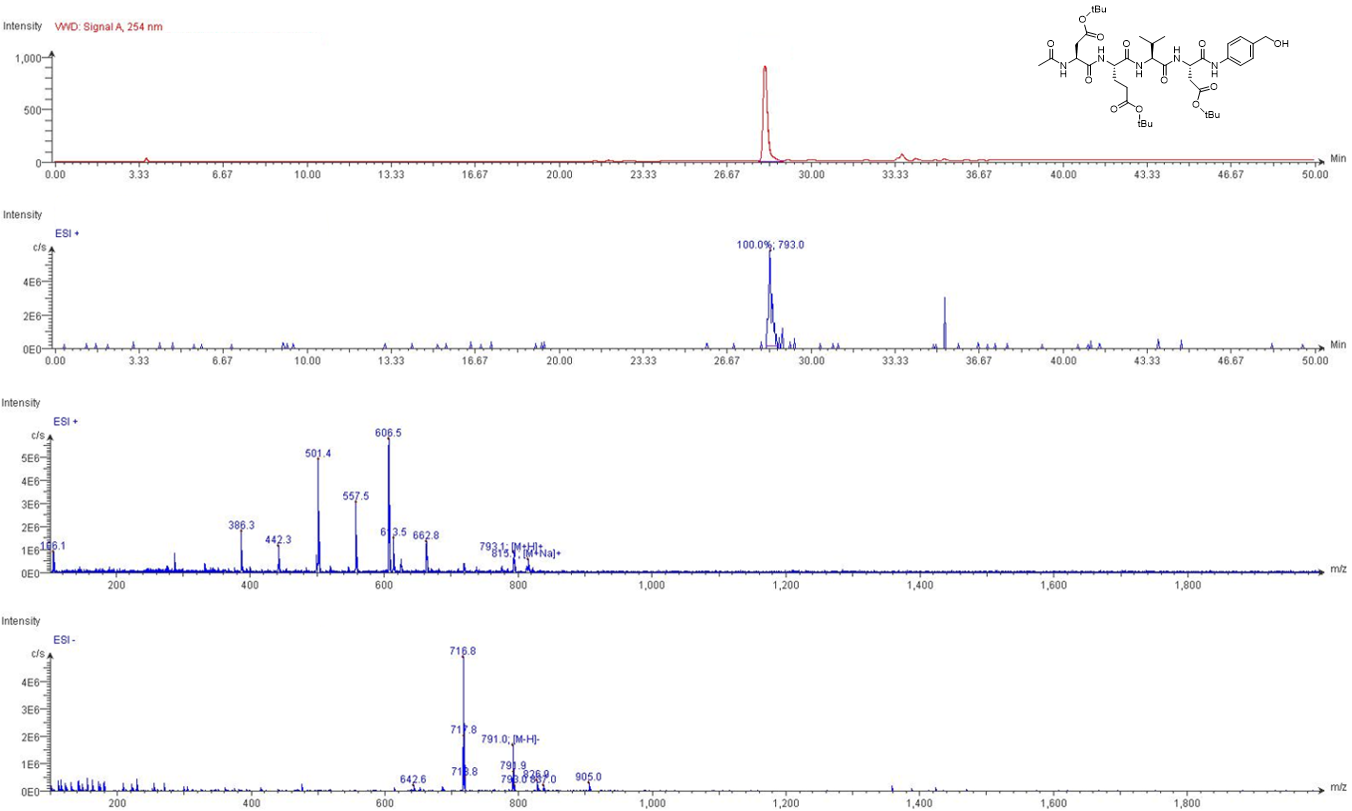


**Figure S4:** LC-MS data for **Ac-DEVD(O*t*Bu)-PABA-OH**.


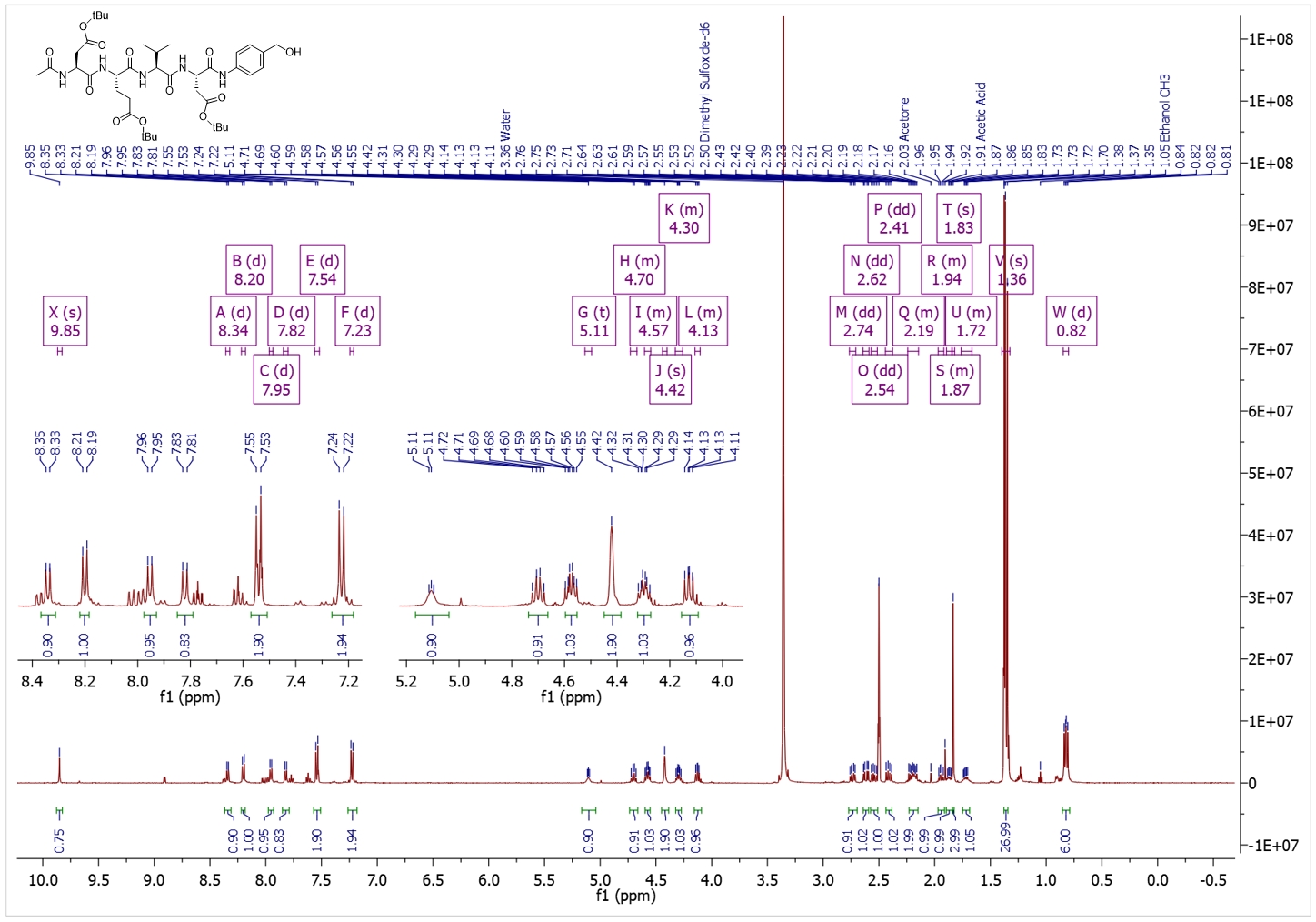


**Figure S5:** ^1^H NMR spectrum of **Ac-DEVD(O*t*Bu)-PABA-OH** in DMSO-*d*_6_.


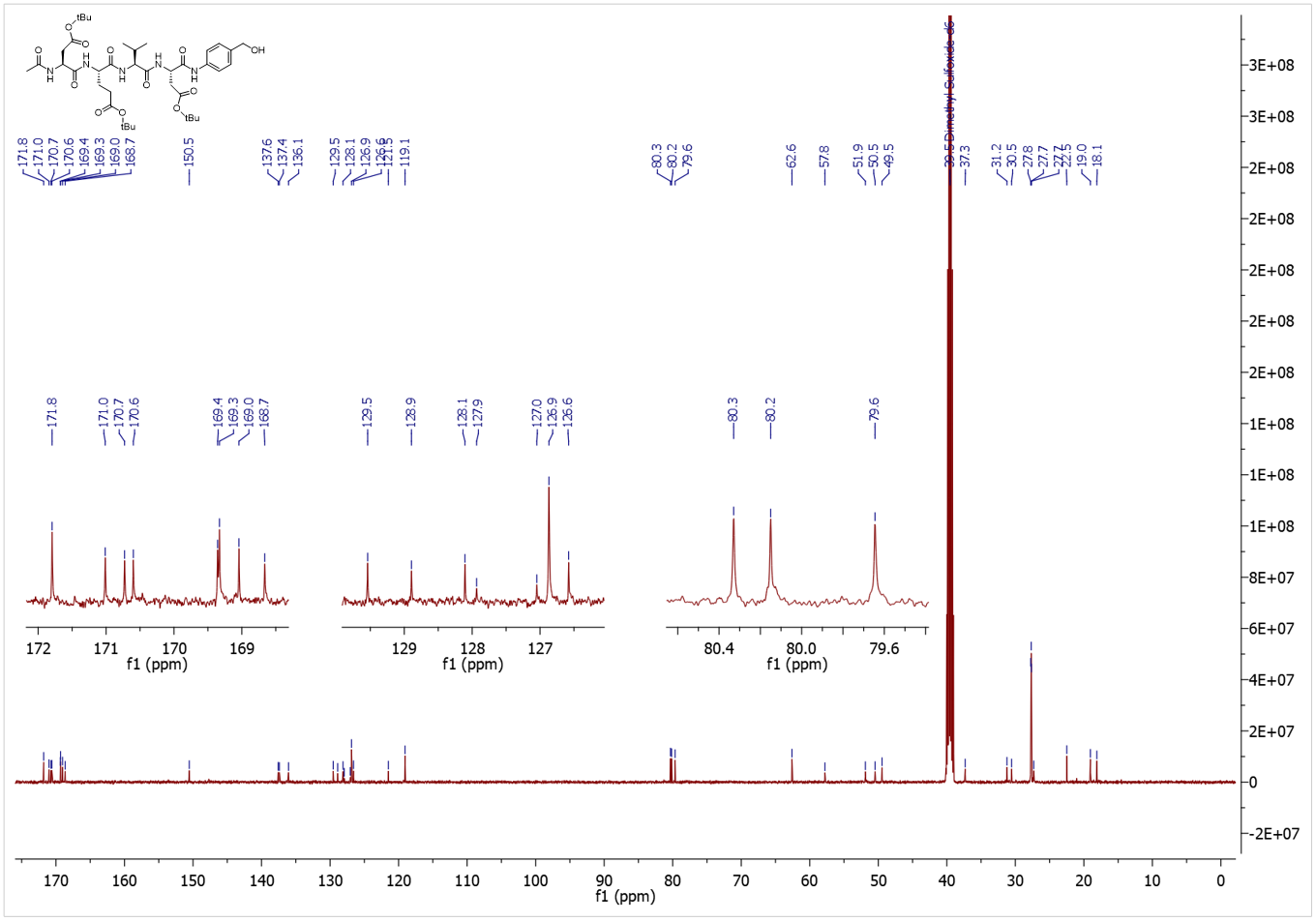


**Figure S6:** ^13^C NMR spectrum of **Ac-DEVD(O*t*Bu)-PABA-OH** in DMSO-*d*_6_.


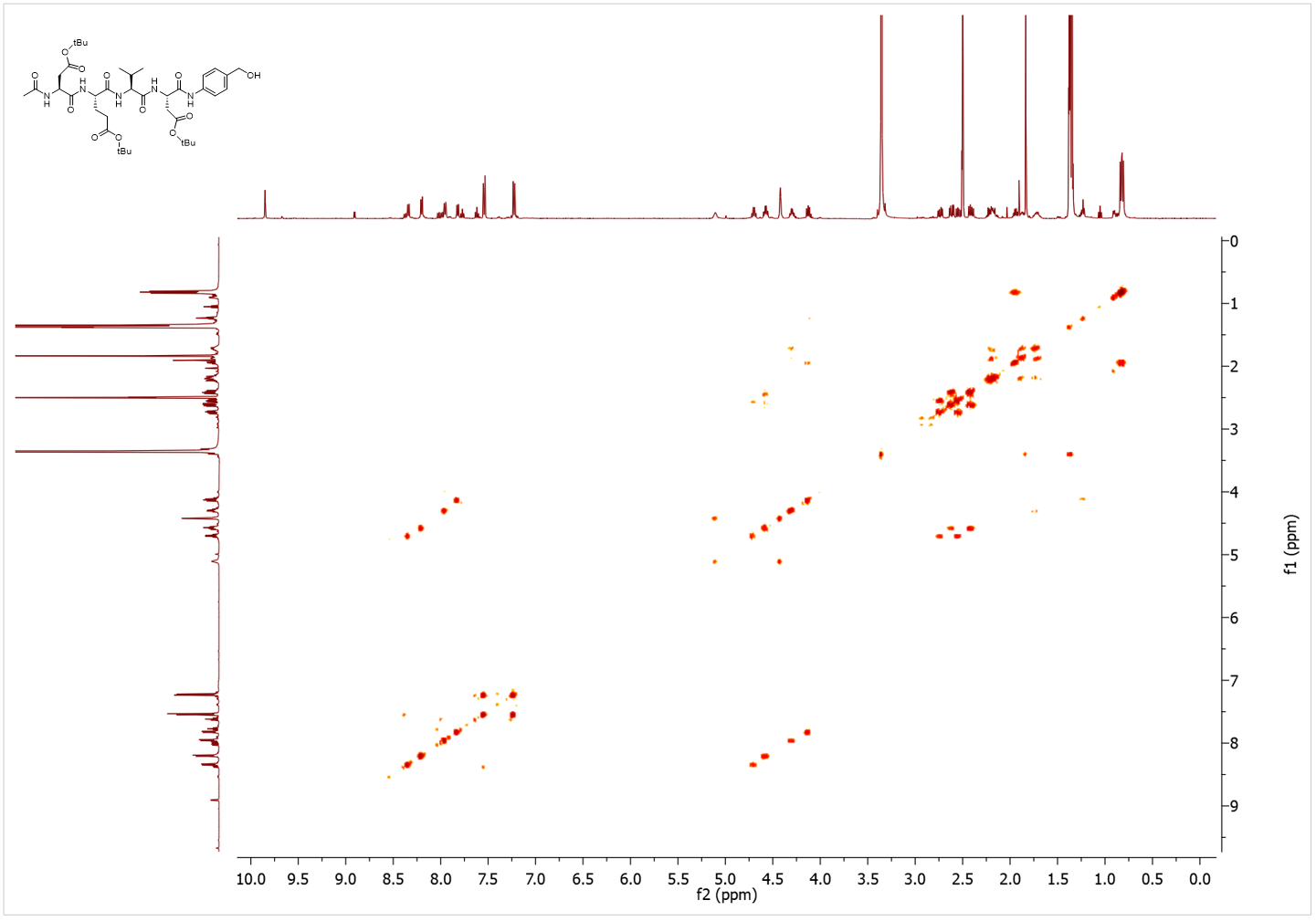


**Figure S7:** COSY spectrum of **Ac-DEVD(O*t*Bu)-PABA-OH** in DMSO-*d*_6_.


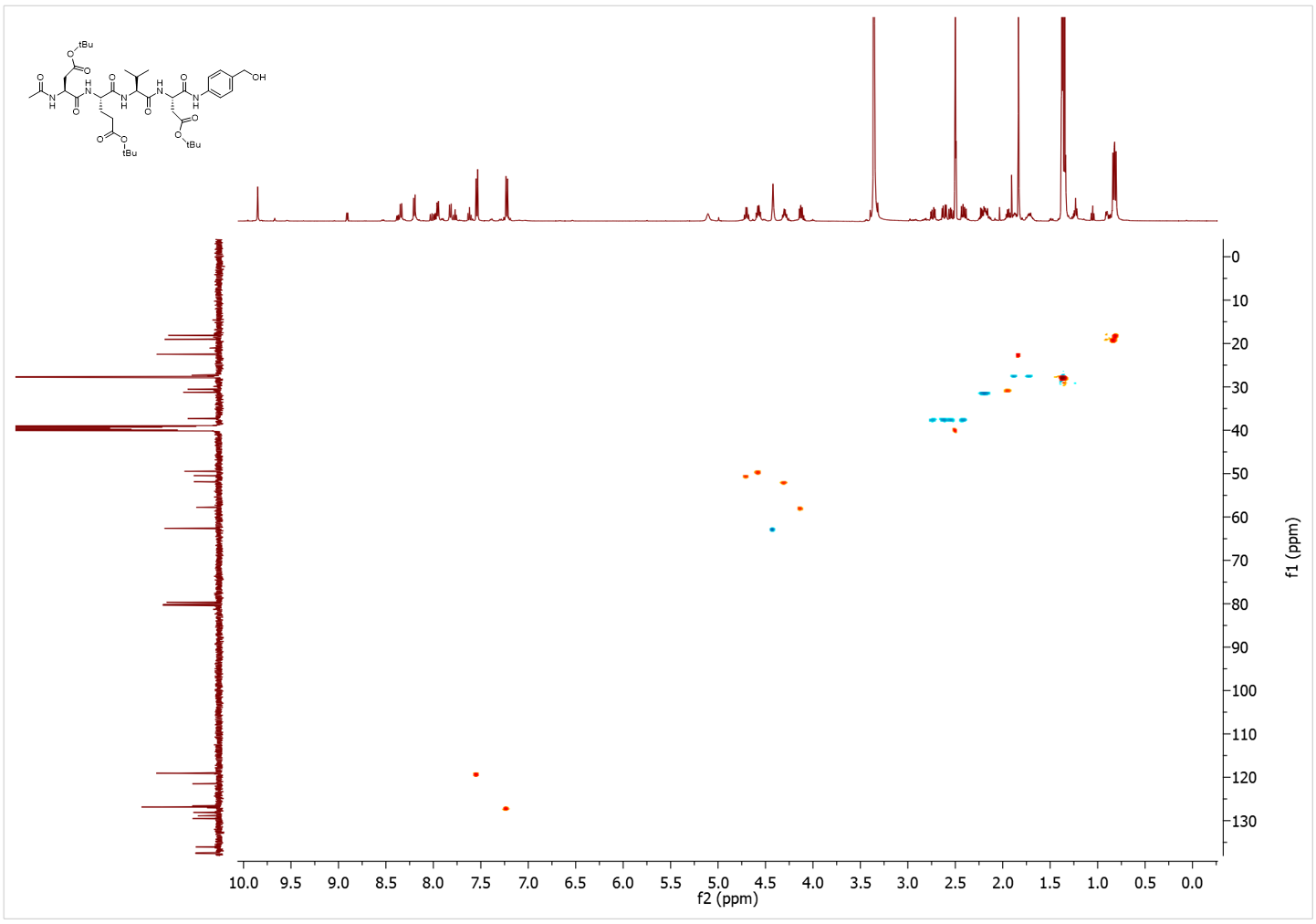


**Figure S8:** HSQC spectrum of **Ac-DEVD(O*t*Bu)-PABA-OH** in DMSO-*d*_6_.


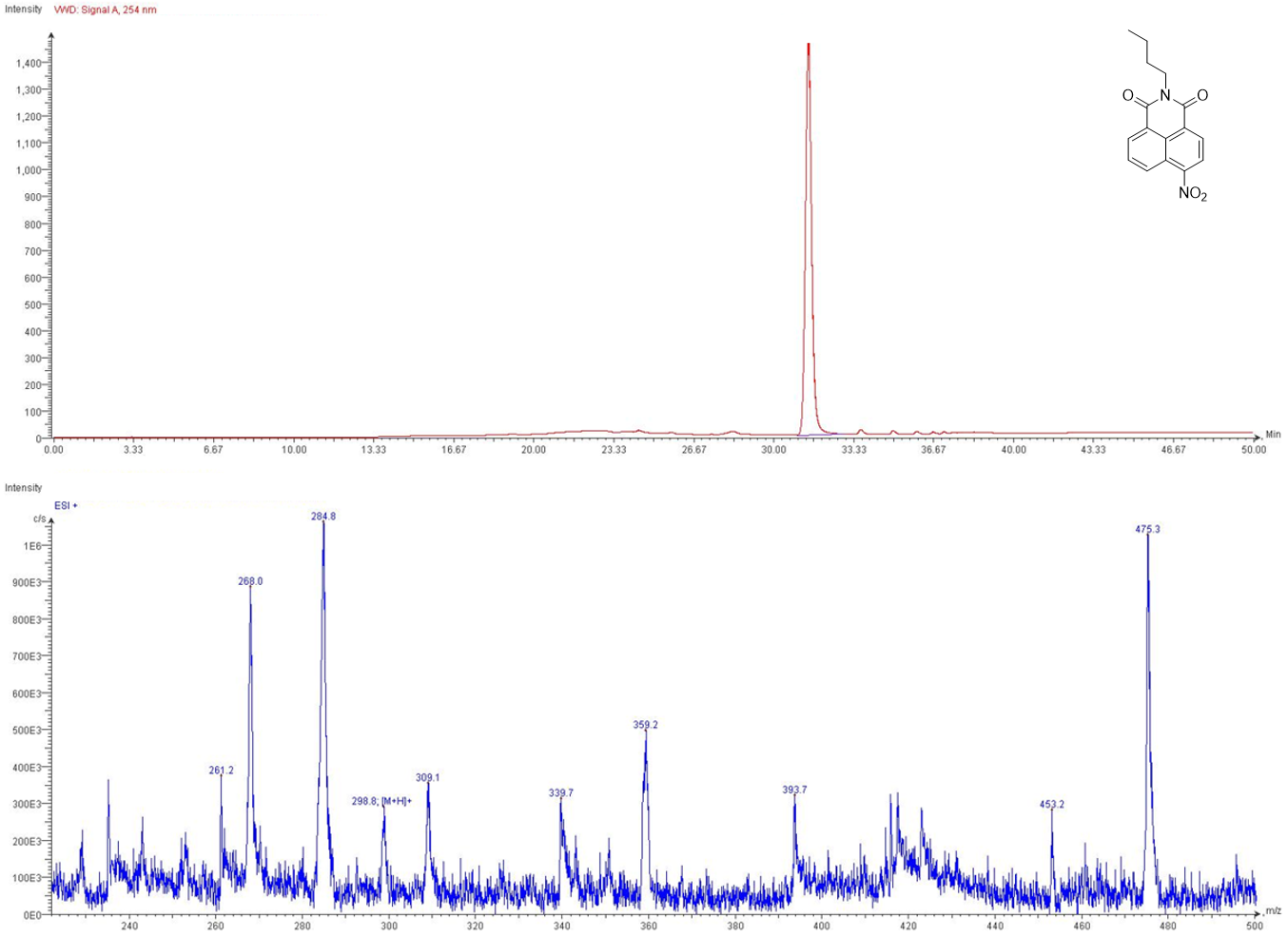


**Figure S9:** LC-MS data for **4-nitro-*N*-butyl-1,8-naphthalimide**.


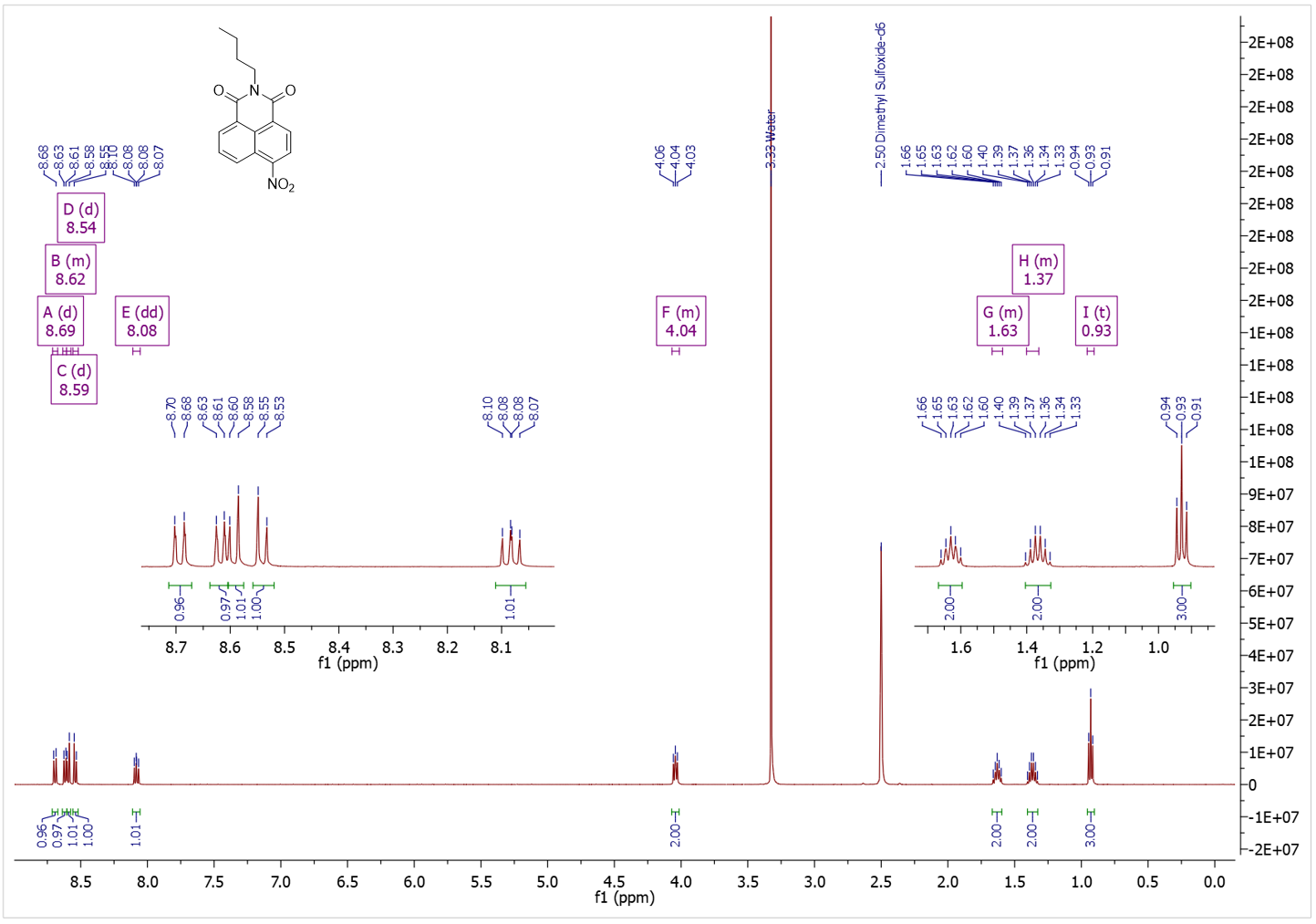


**Figure S10:** ^1^H NMR Spectrum of **4-nitro-*N*-butyl-1,8-naphthalimide** in DMSO-*d*_6_.


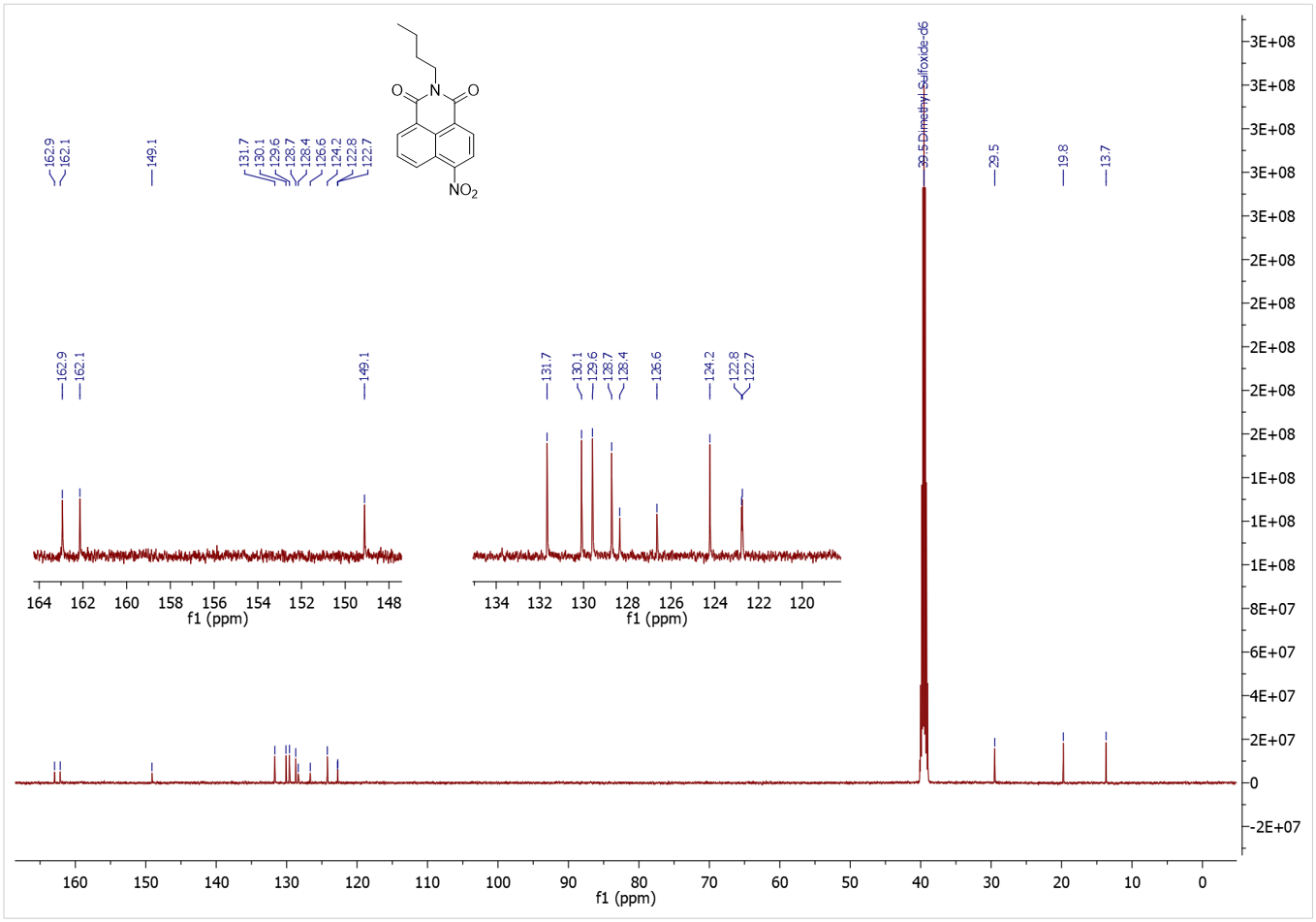


**Figure S11:** ^13^C NMR Spectrum of **4-nitro-*N*-butyl-1,8-naphthalimide** in DMSO-*d*_6_.


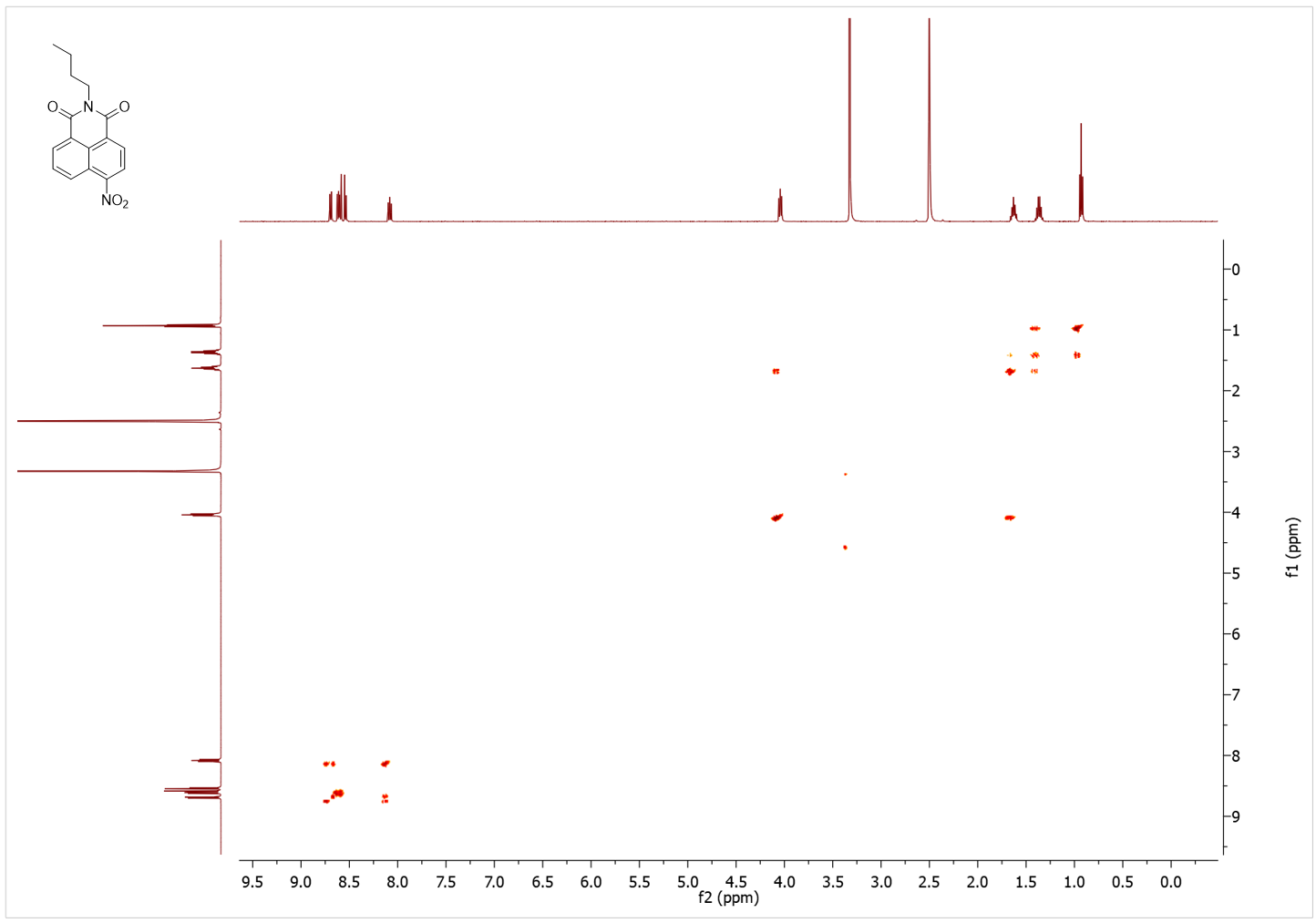


**Figure S12:** COSY Spectrum of **4-nitro-*N*-butyl-1,8-naphthalimide** in DMSO-*d*_6_.


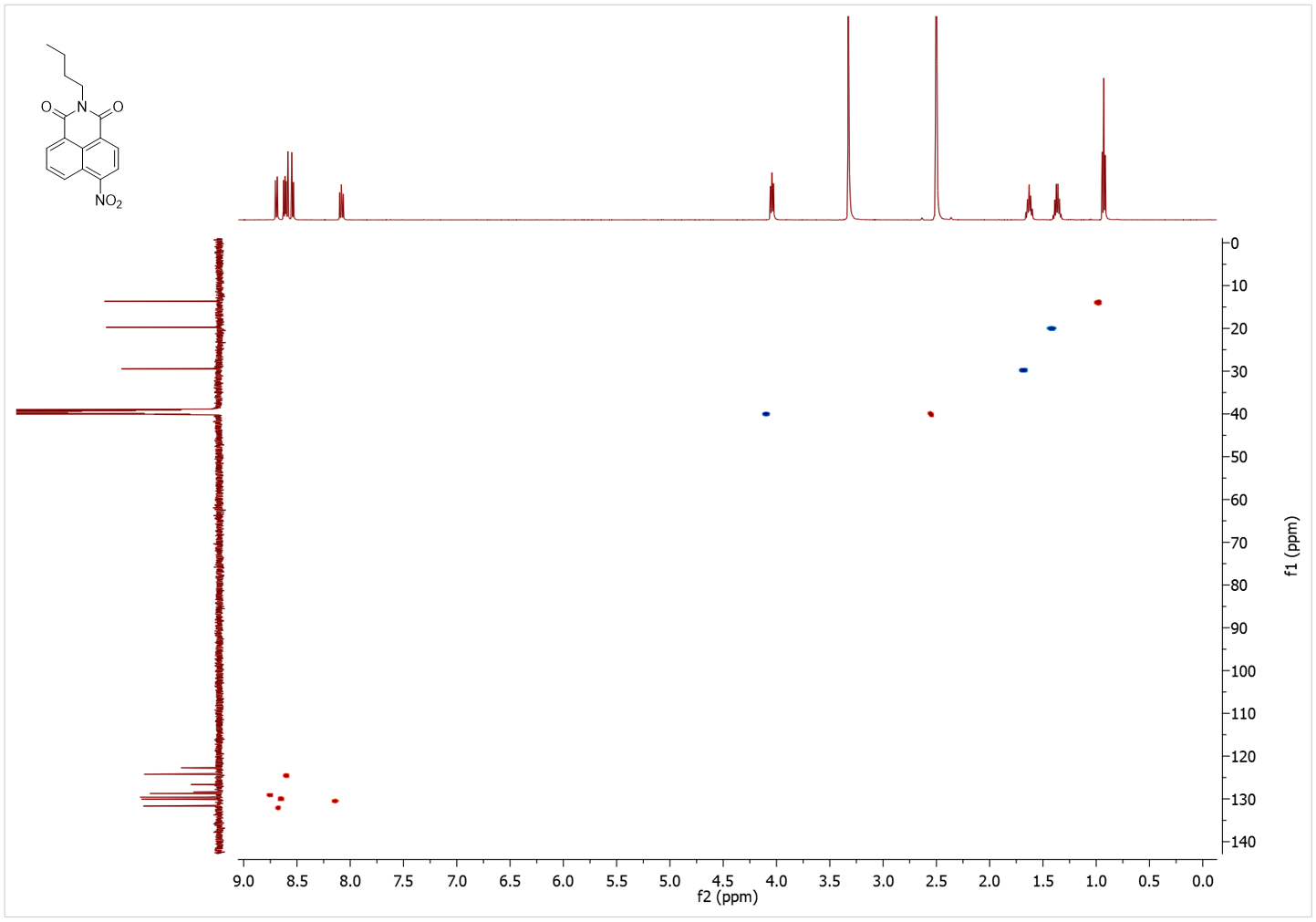


**Figure S13:** HSQC Spectrum of **4-nitro-*N*-butyl-1,8-naphthalimide** in DMSO-*d*_6_.


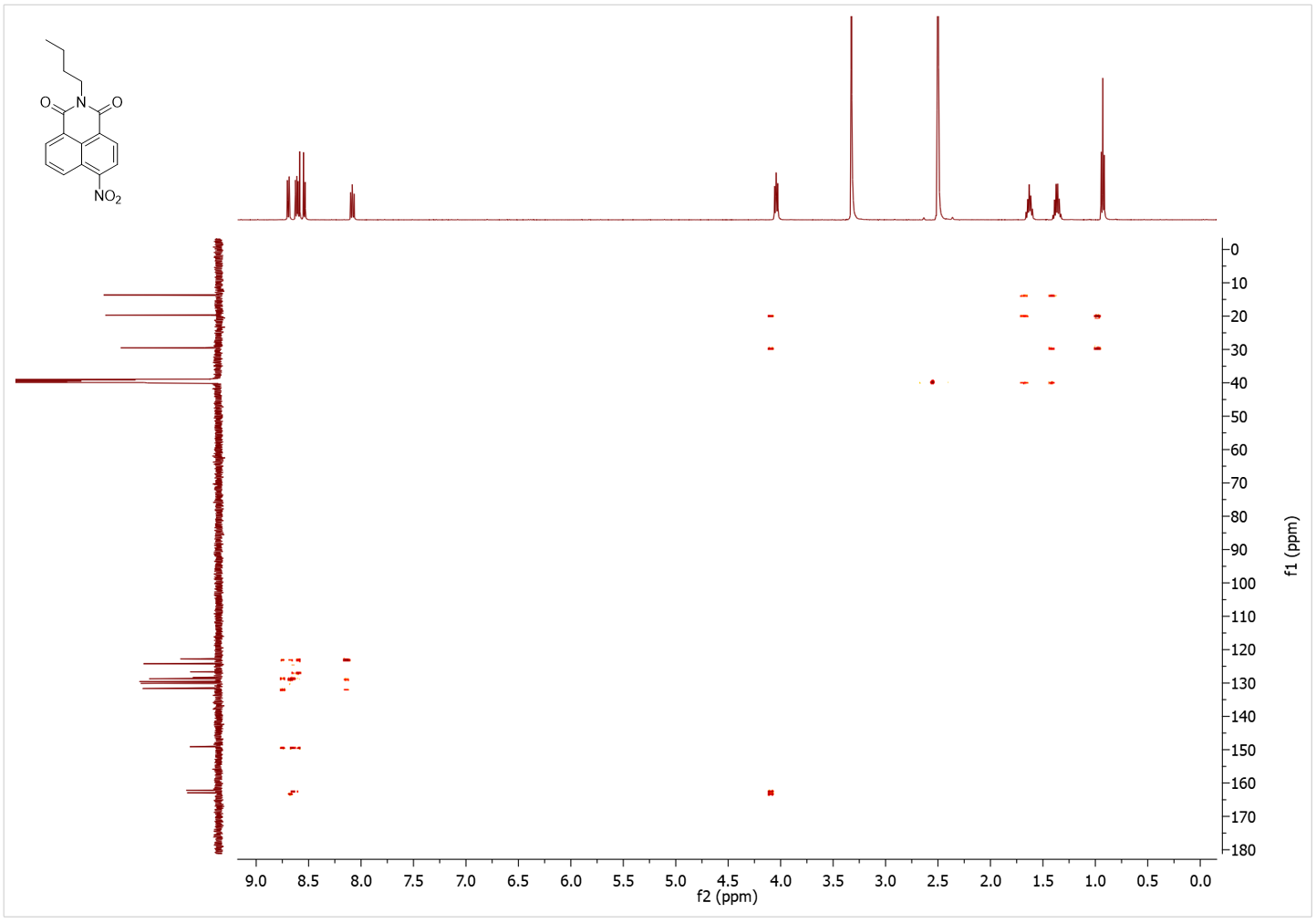


**Figure S14:** HMBC Spectrum of **4-nitro-*N*-butyl-1,8-naphthalimide** in DMSO-*d*_6_.


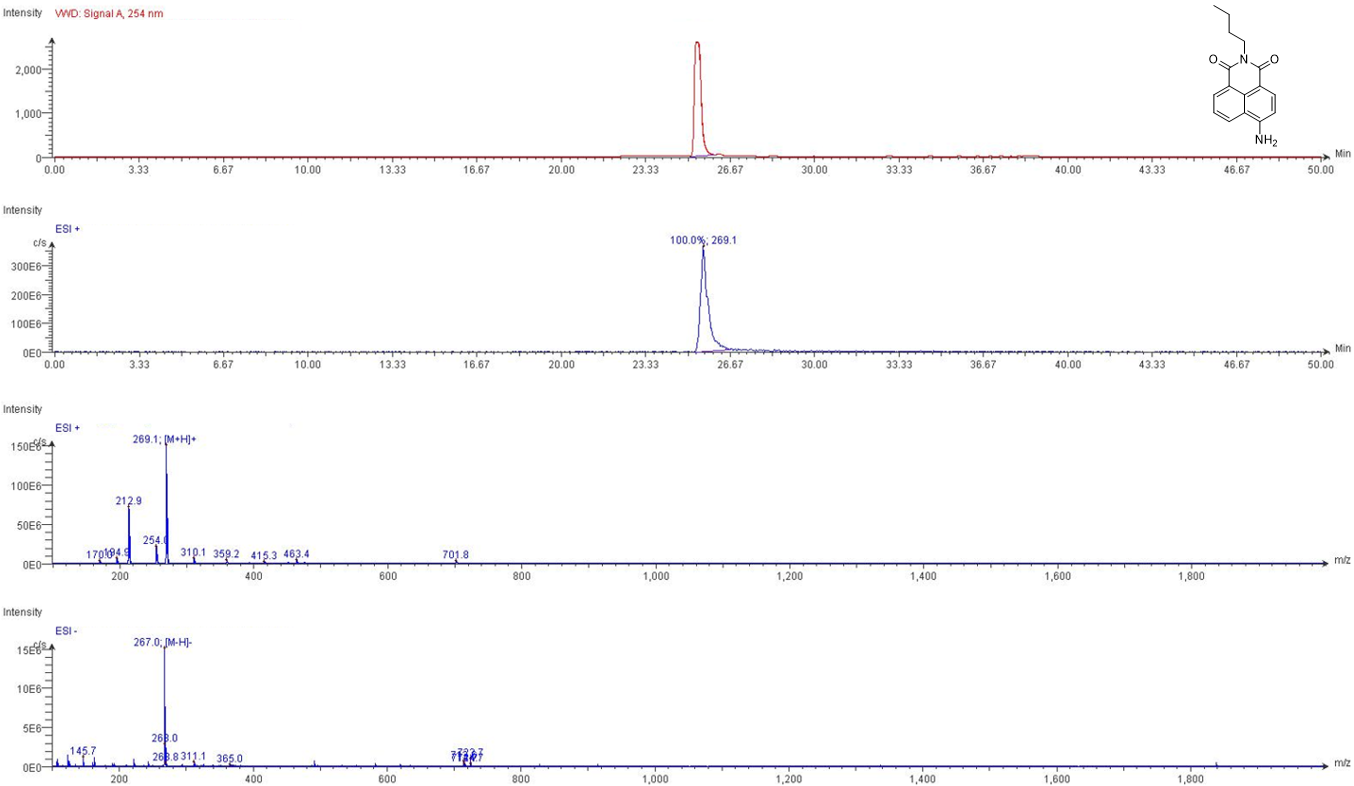


**Figure S15:** LC-MS data for **4-amino-*N*-butyl-1,8-naphthalimide**.


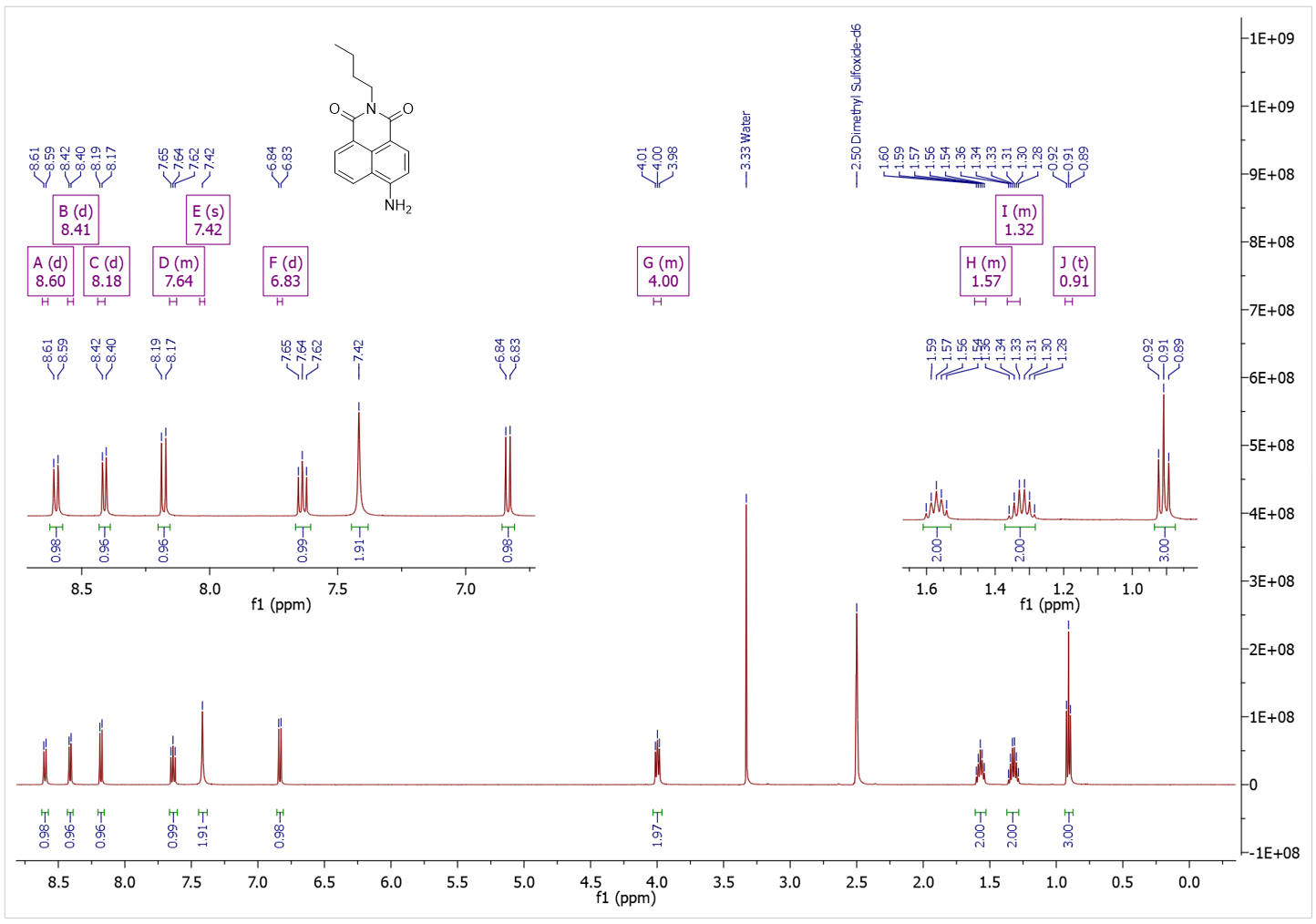


**Figure S16:** ^1^H NMR spectrum of **4-amino-*N*-butyl-1,8-naphthalimide** in DMSO-*d*_6_.


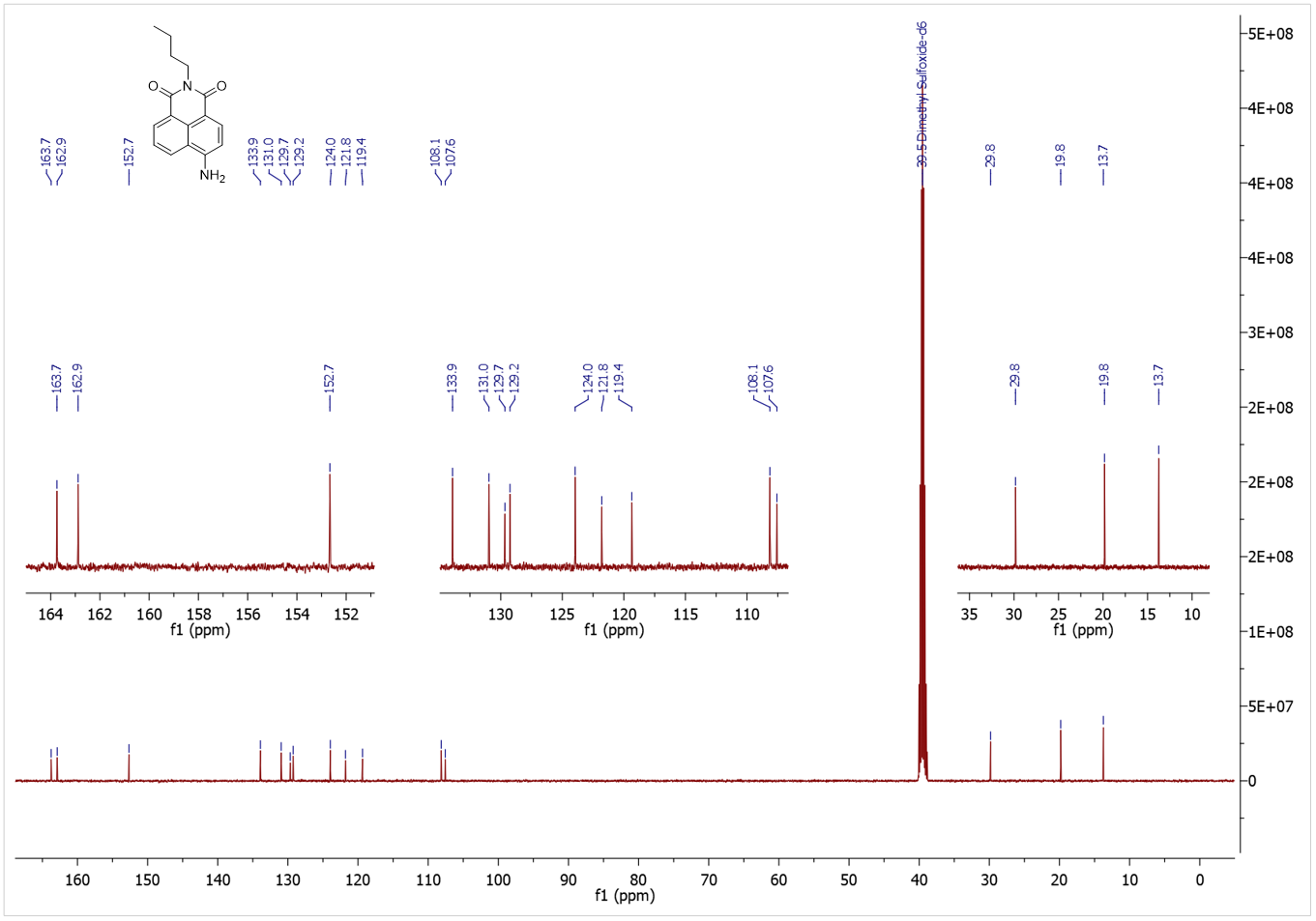


**Figure S17:** ^13^C NMR spectrum of **4-amino-*N*-butyl-1,8-naphthalimide** in DMSO-*d*_6_.


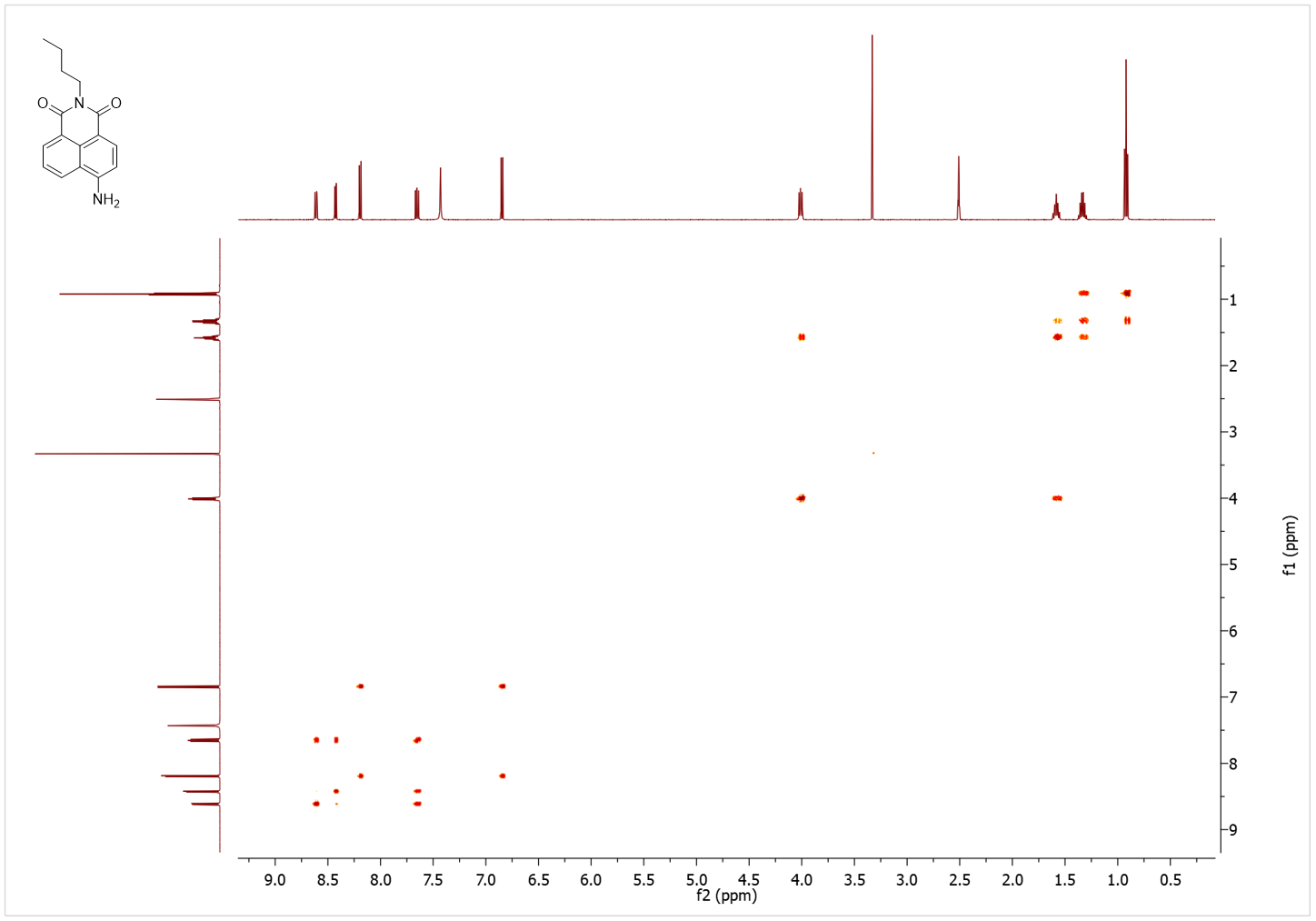


**Figure S18:** COSY spectrum of **4-amino-*N*-butyl-1,8-naphthalimide** in DMSO-*d*_6_.


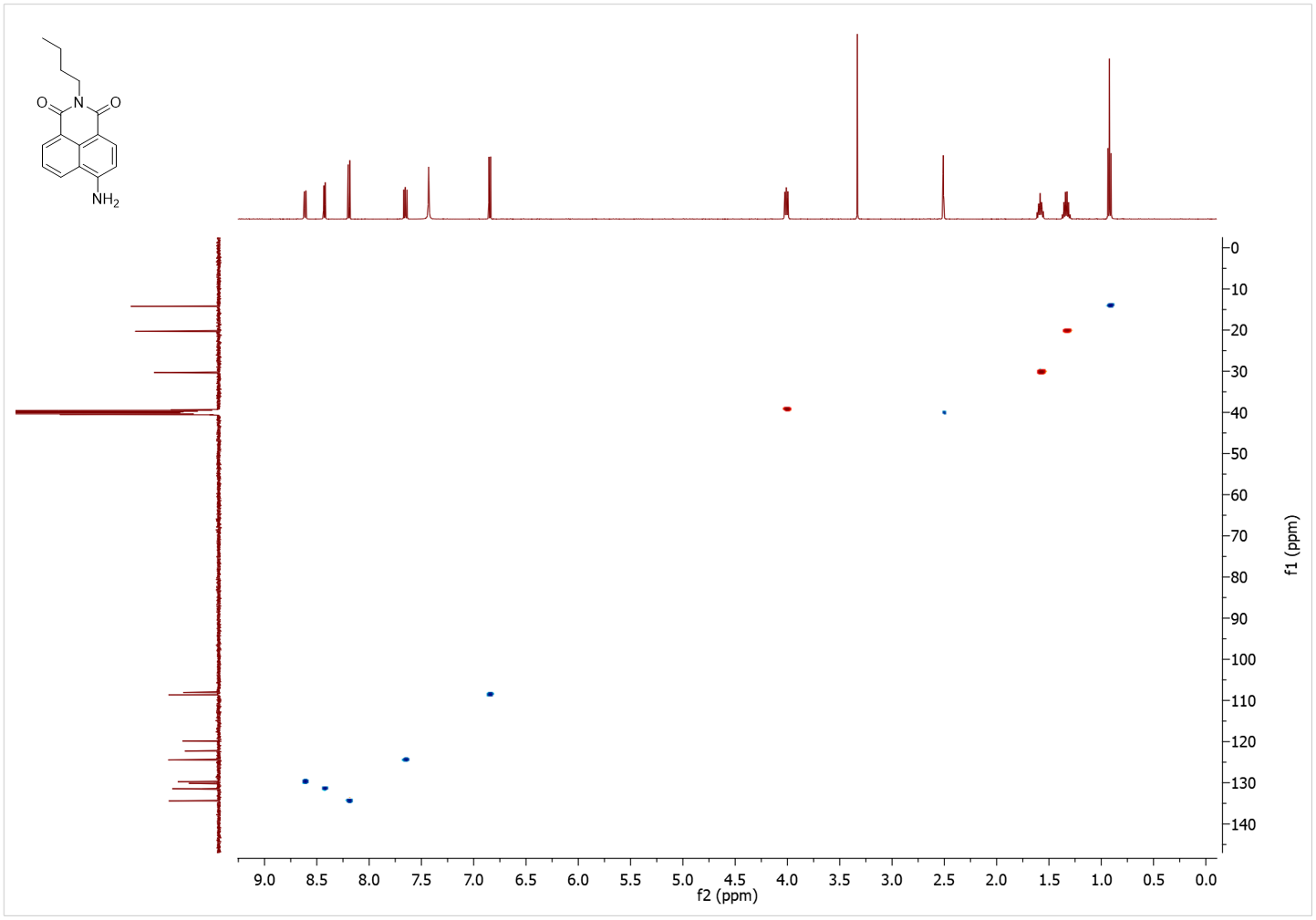


**Figure S19:** HSQC spectrum of **4-amino-*N*-butyl-1,8-naphthalimide** in DMSO-*d*_6_.


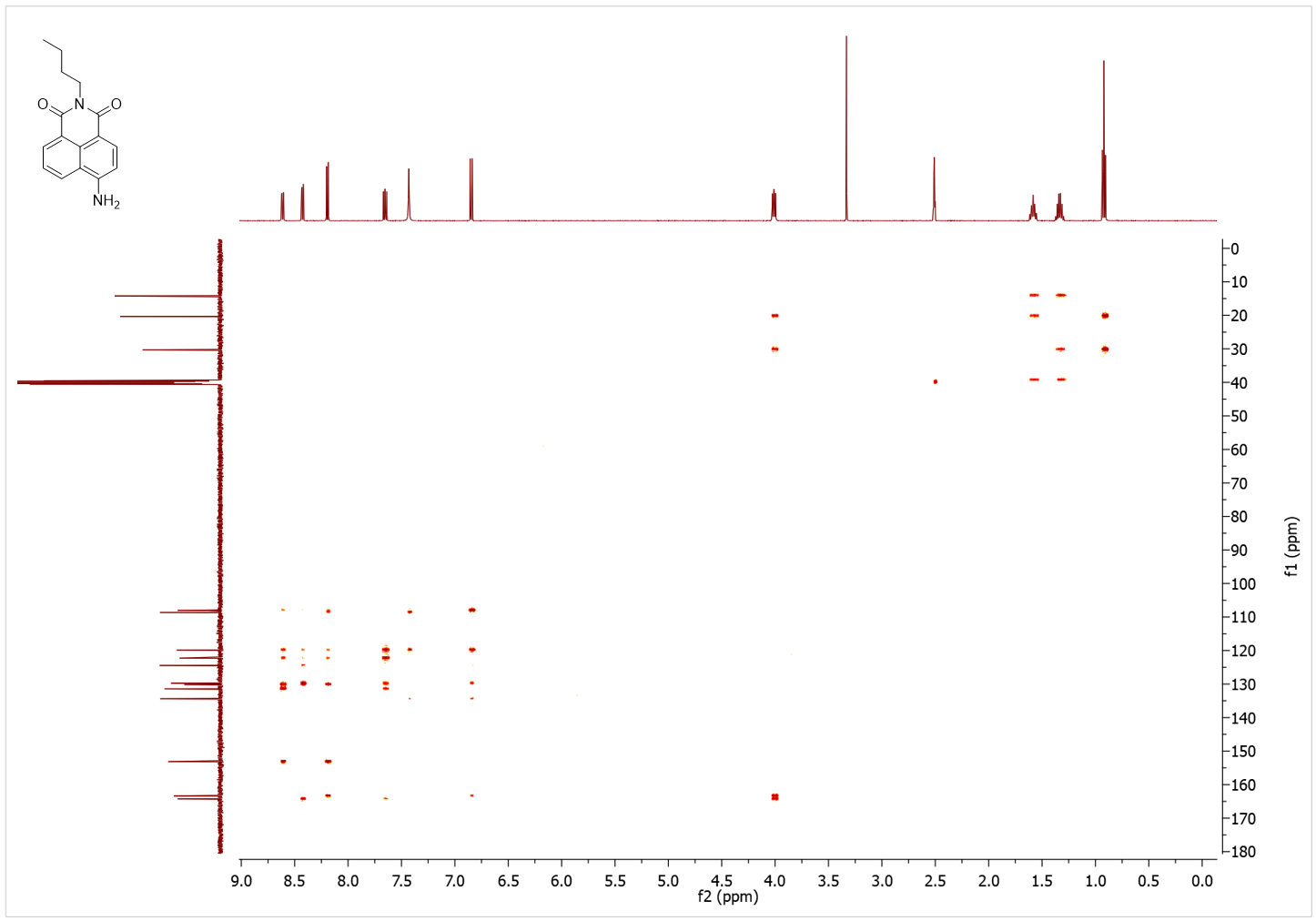


**Figure S20:** HMBC spectrum of **4-amino-*N*-butyl-1,8-naphthalimide** in DMSO-*d*_6_.


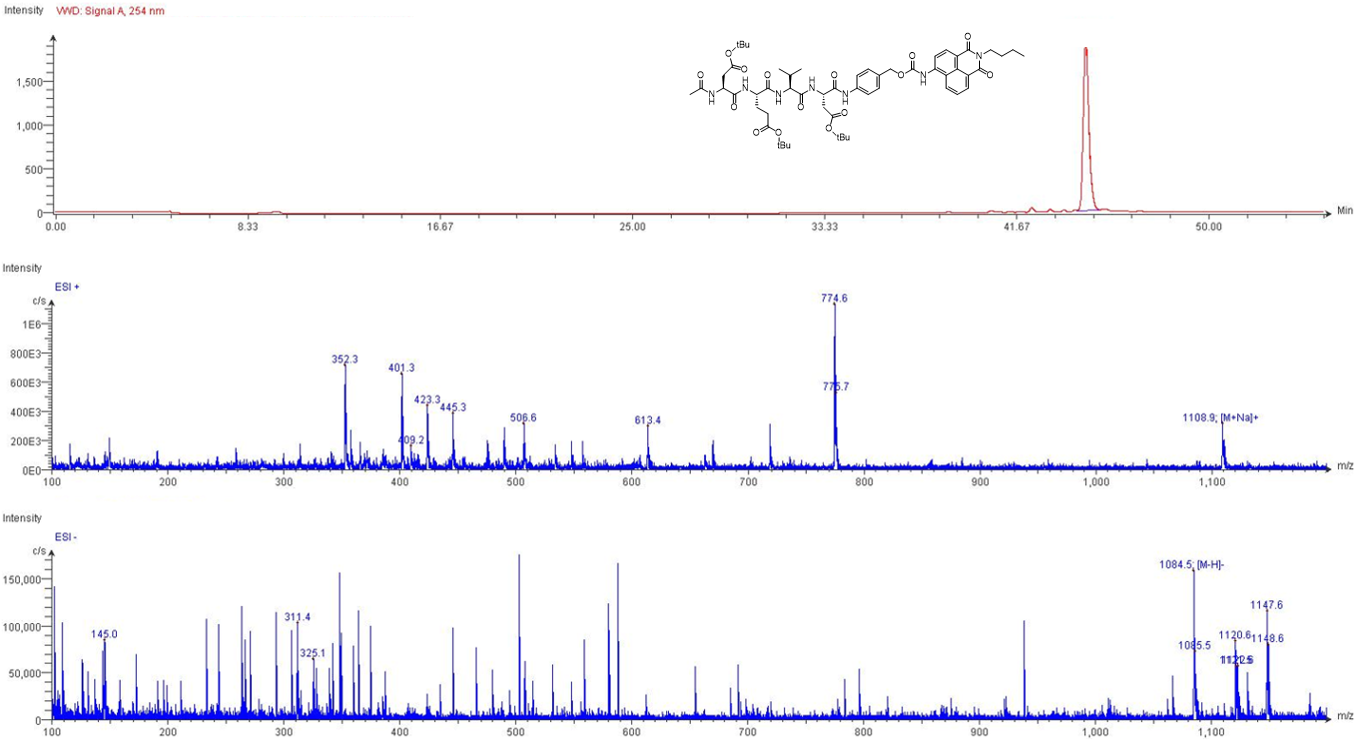


**Figure S21:** LC-MS data for **Ac-DEVD(O*t*Bu)-PABC-Naph**.


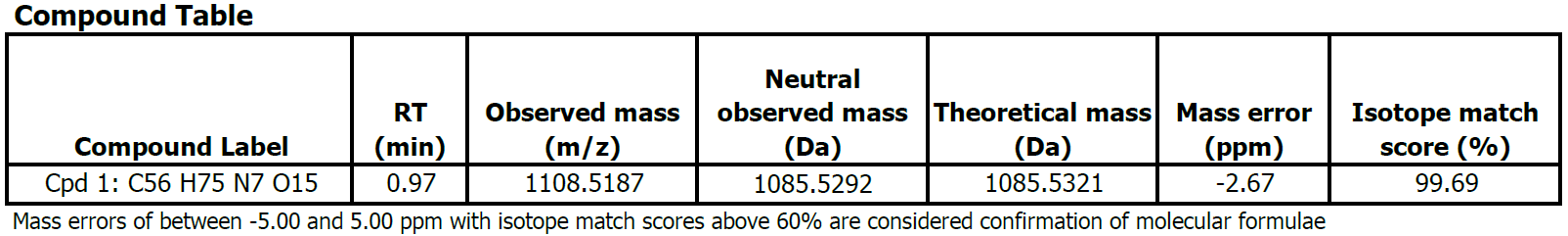

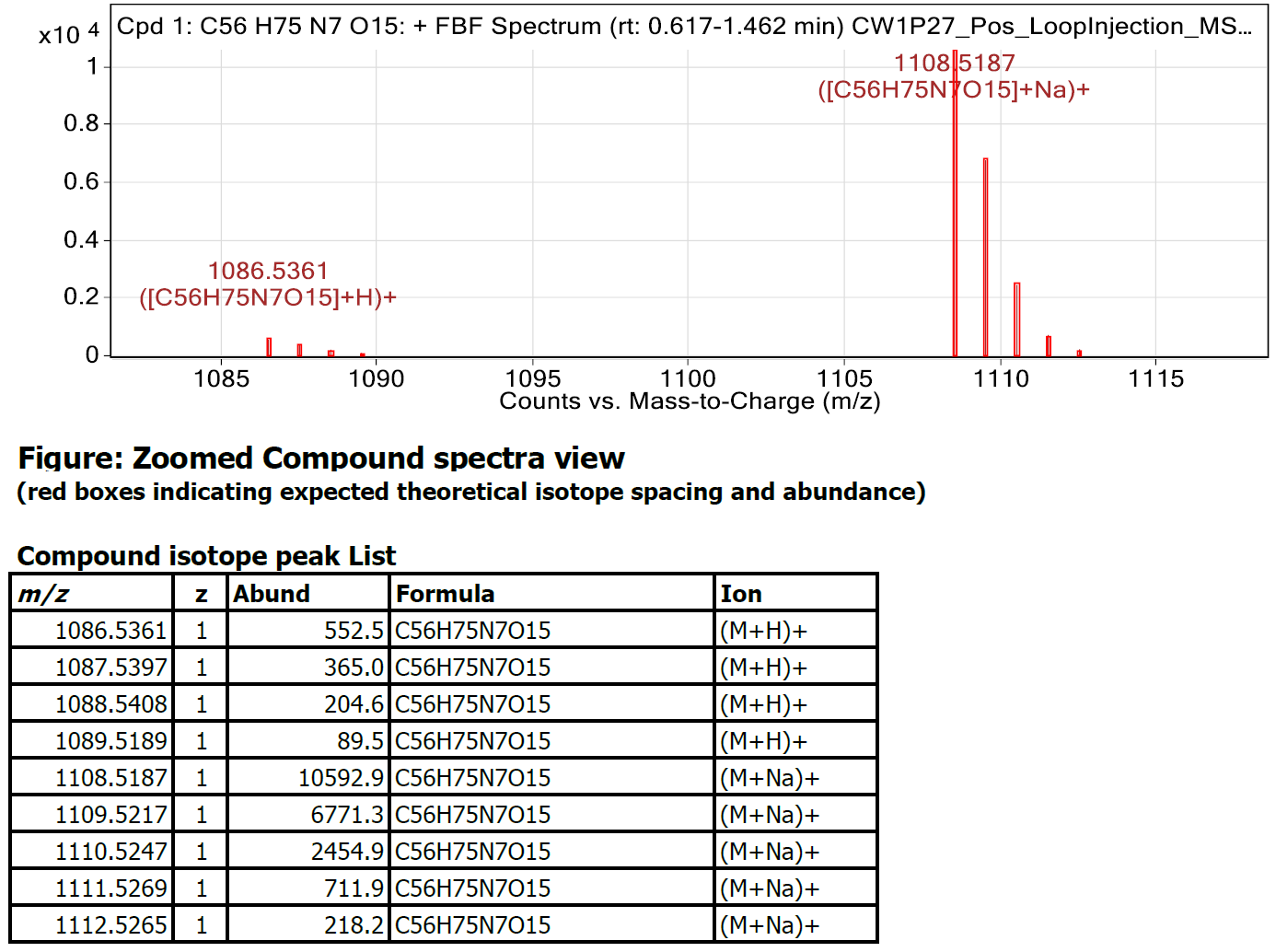


**Figure S22:** HRMS data for **Ac-DEVD(O*t*Bu)-PABC-Naph**.


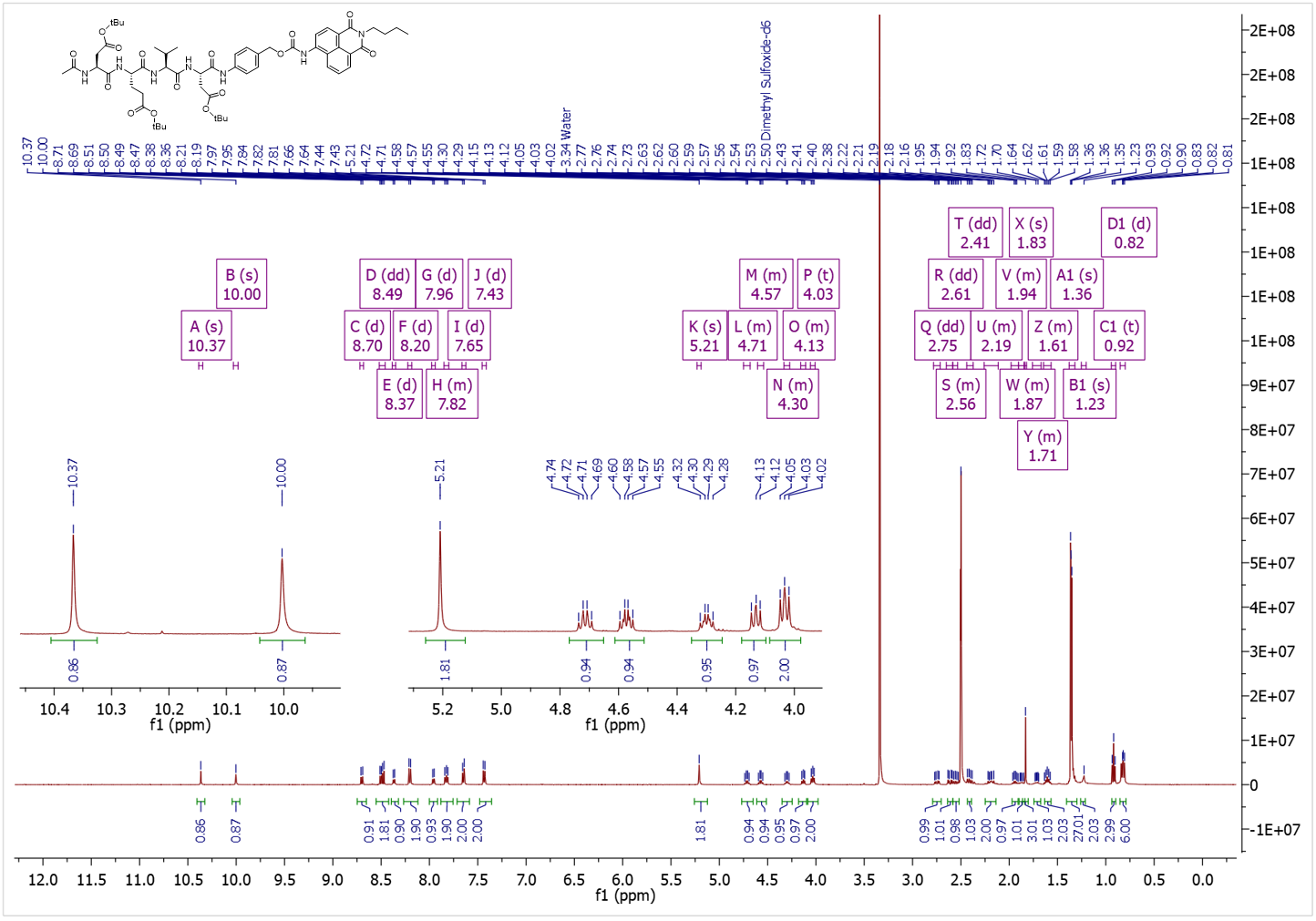


**Figure S23:** ^1^H NMR spectrum of **Ac-DEVD(O*t*Bu)-PABC-Naph** in DMSO-*d*_6_.


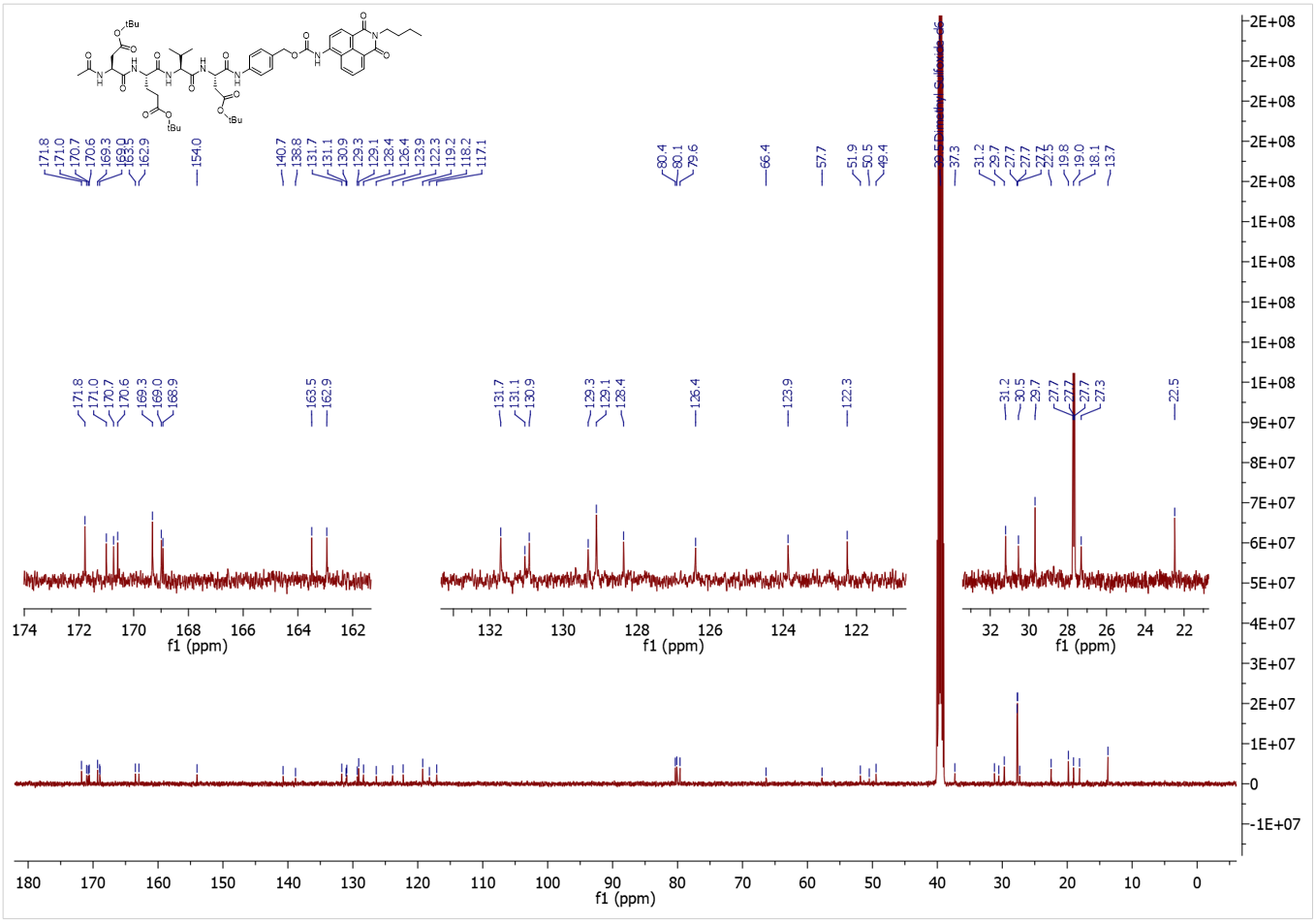


**Figure S24:** ^13^C NMR spectrum of **Ac-DEVD(O*t*Bu)-PABC-Naph** in DMSO-*d*_6_.


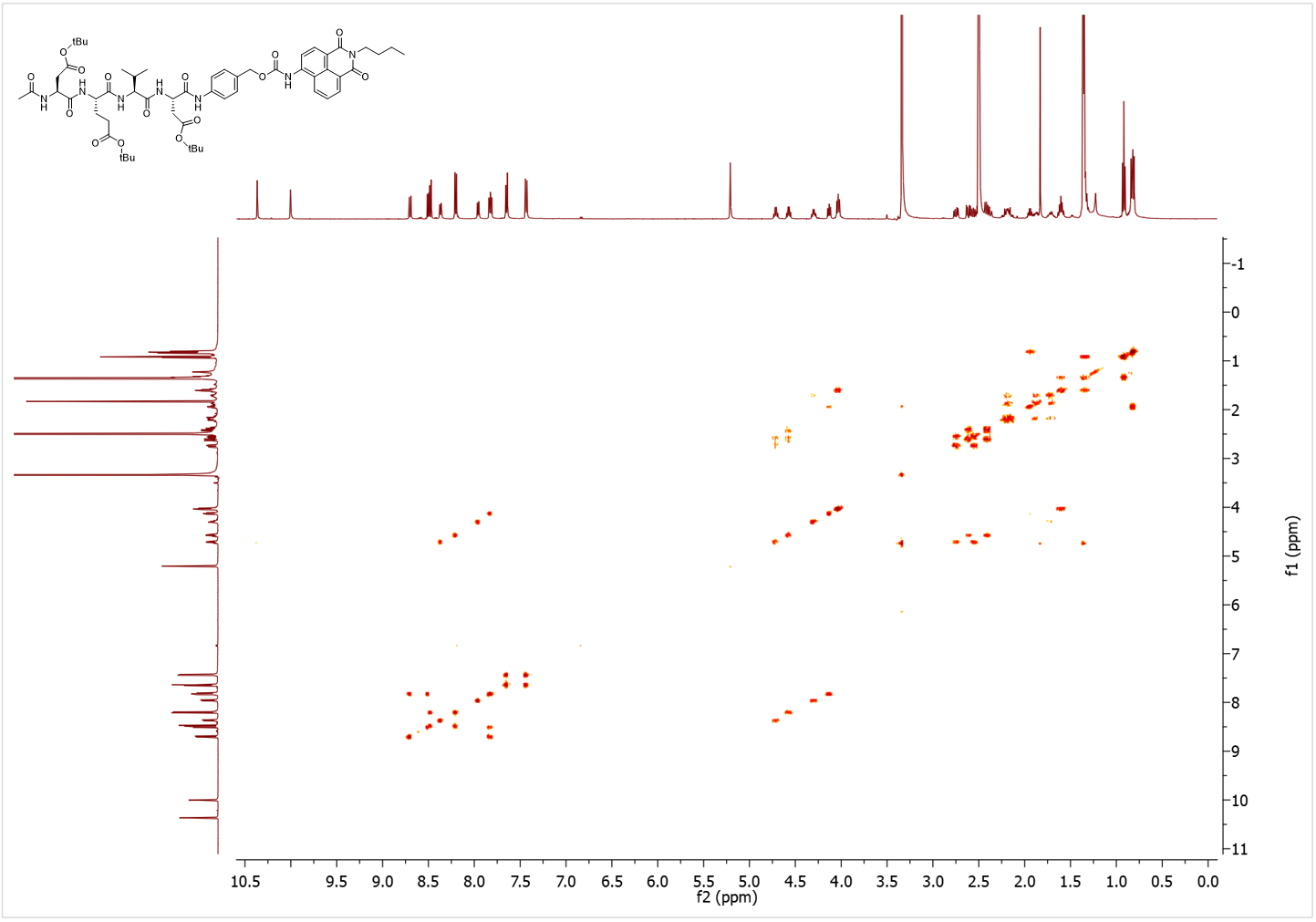


**Figure S25:** COSY spectrum of **Ac-DEVD(O*t*Bu)-PABC-Naph** in DMSO-*d*_6_.


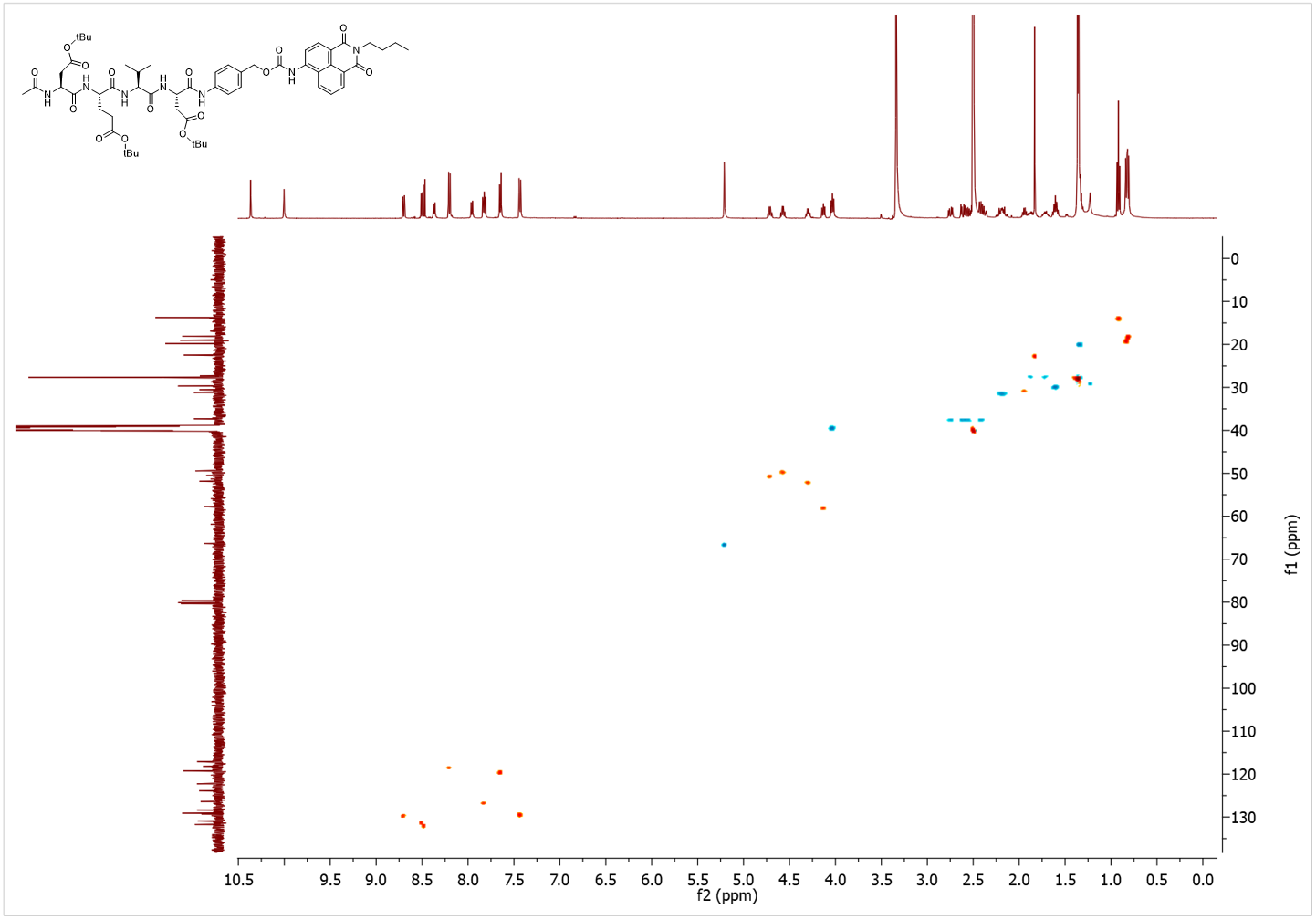


**Figure S26:** HSQC spectrum of **Ac-DEVD(O*t*Bu)-PABC-Naph** in DMSO-*d*_6_.


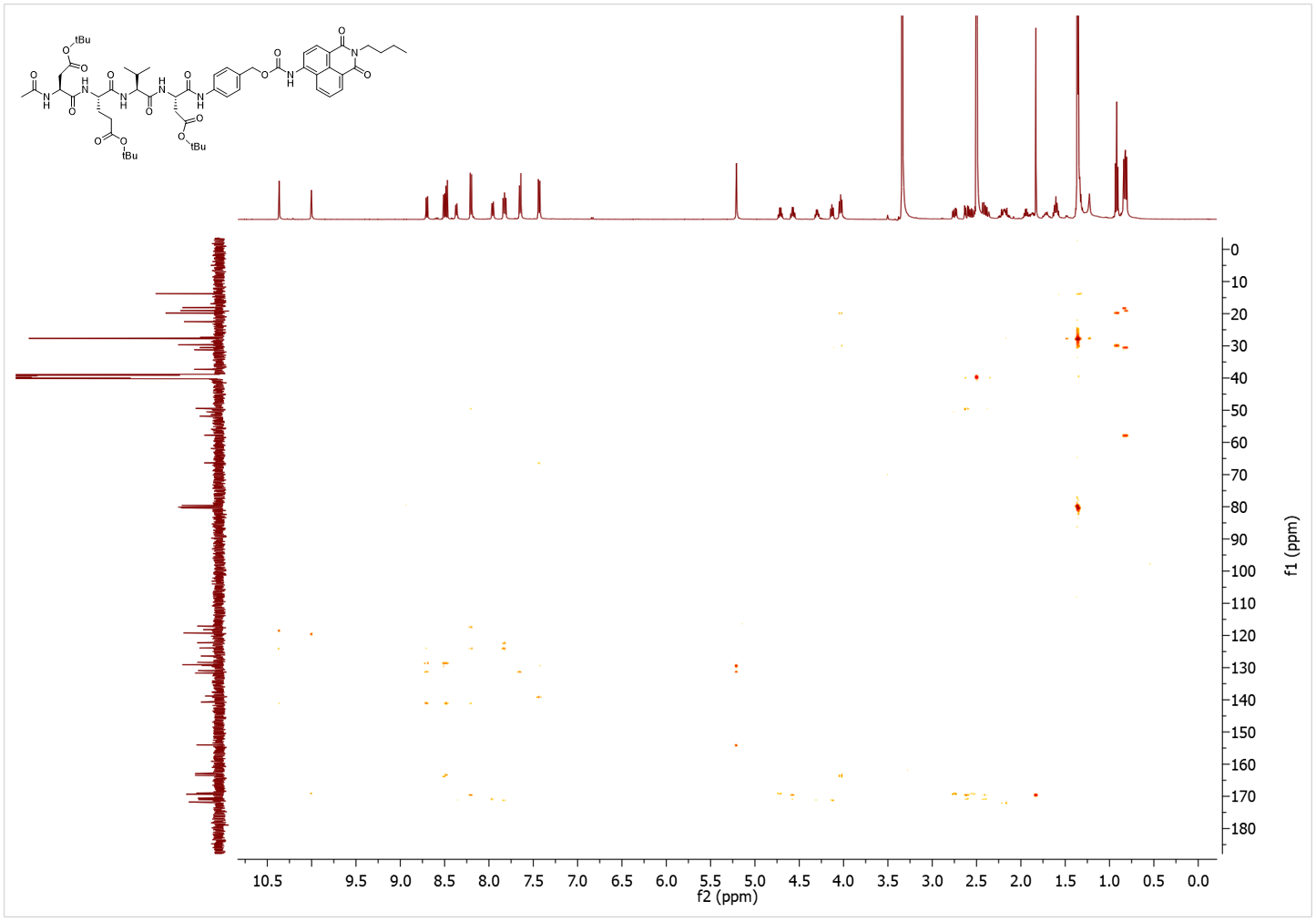


**Figure S27:** HMBC spectrum of **Ac-DEVD(O*t*Bu)-PABC-Naph** in DMSO-*d*_6_.


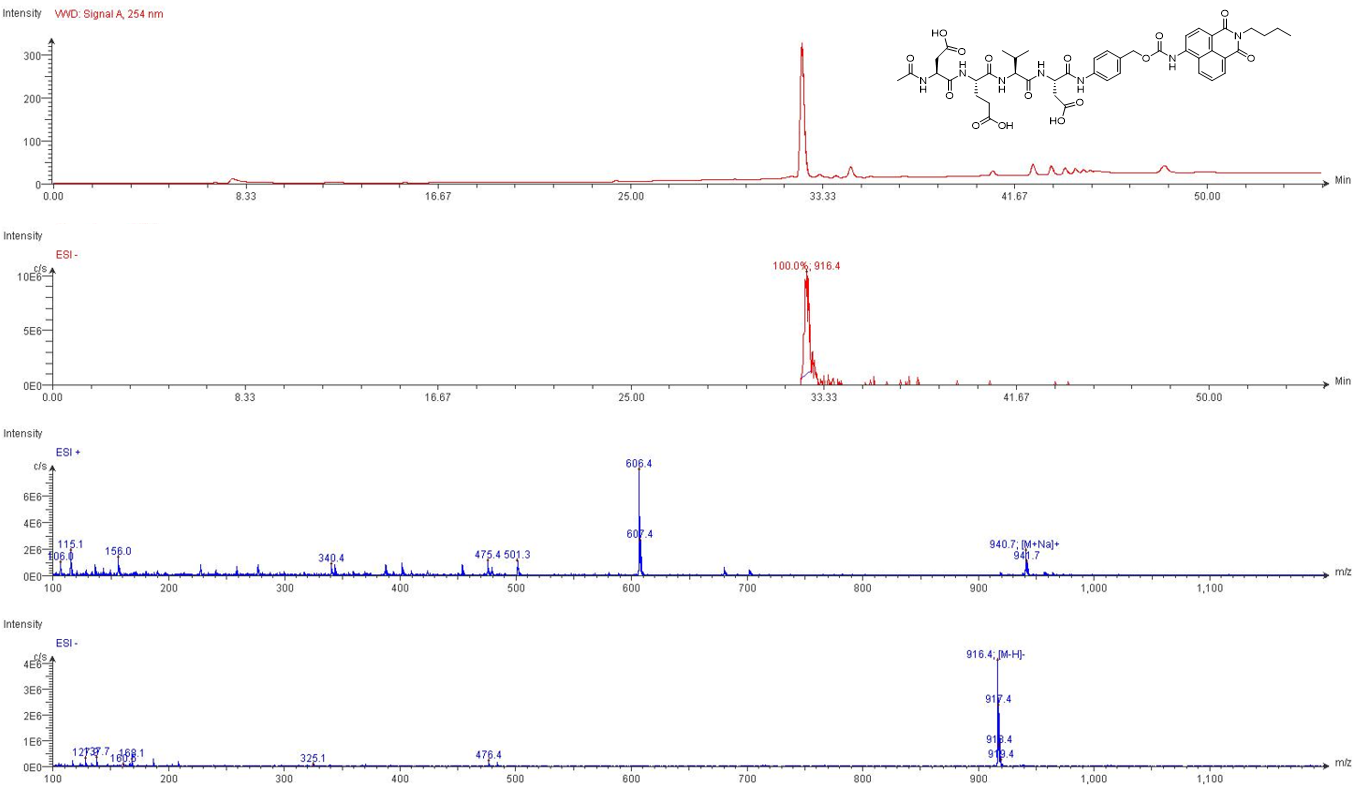


**Figure S28:** LC-MS data for **Ac-DEVD-PABC-Naph**.


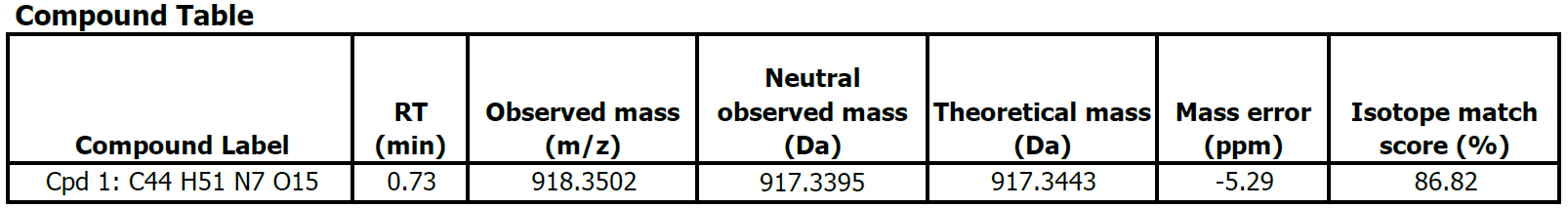

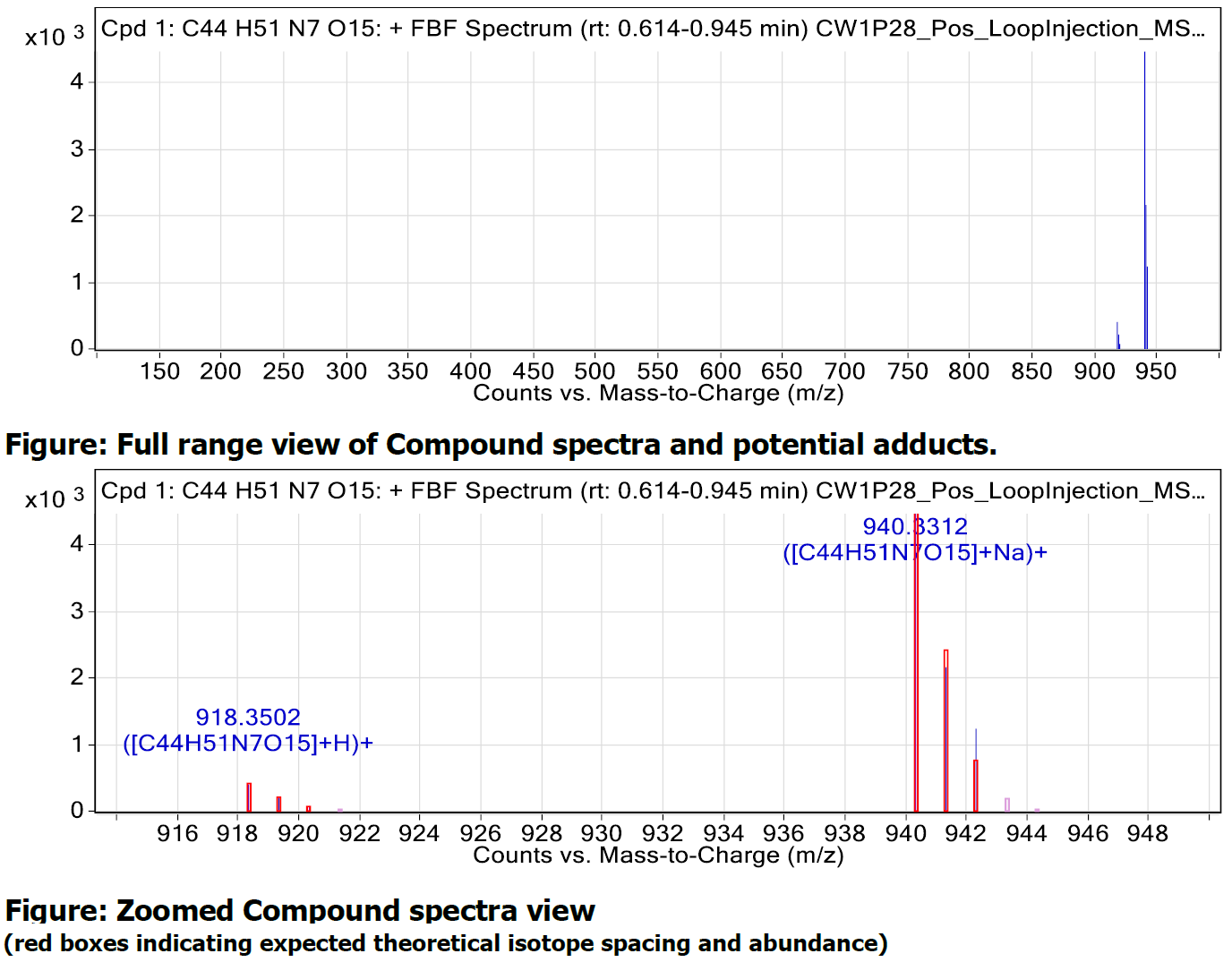

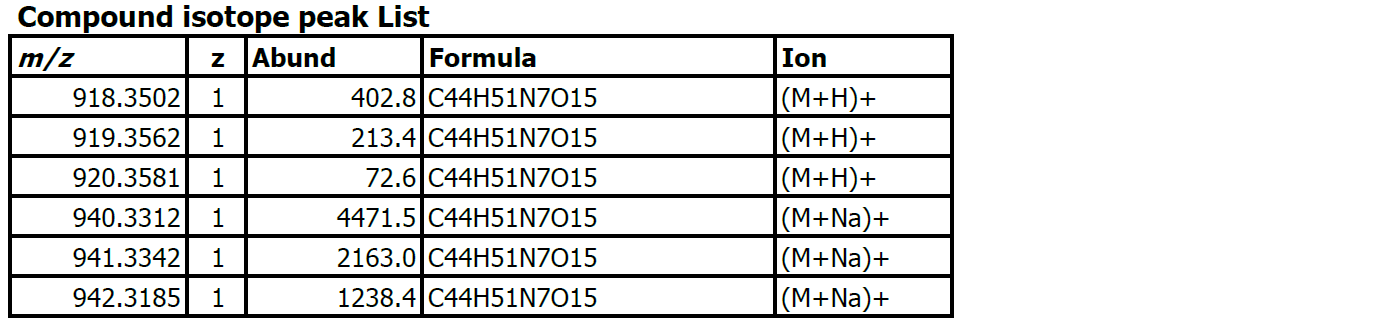


**Figure S29:** HRMS data for **Ac-DEVD-PABC-Naph**.

# Spectroscopic Data

**Figure S30:** Absorbance spectra of Ac-DEVD-PABC-Naph before and after Caspase-3.

**Figure S31:** Fluorescence spectrum of Ac-DEVD-PABC-Naph (5 µM) in H_2_O (0.14% DMSO) with increasing concentrations of NaCl (aq.).

**Figure S32:** Calibration curve of fluorescence intensity (λ_max_ = 535 nm) at known concentrations of Caspase-3 (2.5 – 80 ng/ml). The detection limit (3S/m, in which S is the standard deviation of blank measurements, n = 12, and m is the slope of the linear equation) was determined to be 4.96 ng/ml

**Figure S33:** Response kinetics of Caspase-3 (80 ng/ml) towards different concentrations of Ac-DEVD-PABC-Naph over 5 hrs at 25°C.

# References

ATHANASSOPOULOS, P., BARLOS, K., GATOS, D., HATZI, O. & TZAVARA, C. 1995. Application of 2-chlorotrityl chloride in convergent peptide synthesis. *Tetrahedron Letters,* 36**,** 5645-5648.

GUDE, M., RYF, J. & WHITE, P. D. 2002. An accurate method for the quantitation of Fmoc-derivatized solid phase supports. *Letters in Peptide Science,* 9**,** 203-206.

MERCK. 2024. *Peptide Synthesis and Technical Resources* [Online]. Available: <https://www.merckmillipore.com/IE/en/reagents-chemicals-and-labware/novabiochem-learning-center/peptide-and-ht-organic-synthesis-technical-resources/novabiochem-peptide-synthesis/2emb.qB.Fw0AAAFLAyUp.xHi,nav?ReferrerURL=https%3A%2F%2Fwww.google.com%2F&bd=1> [Accessed].

**
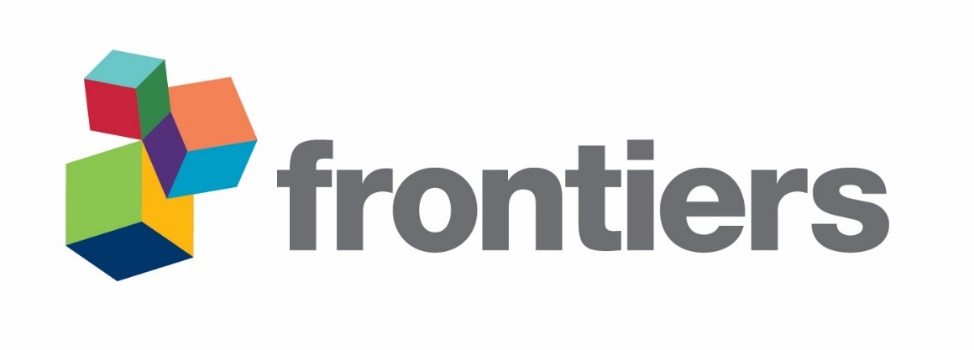
**
